# Lecithin Alleviates Memory Deficits and Muscle Attenuation in Chinese Older Adults and SAMP8 Mice

**DOI:** 10.1002/advs.202405222

**Published:** 2025-05-09

**Authors:** Xianyun Wang, Dajun Li, Xiao Ying Li, Weizhao Lu, Huini Ding, Chengyan Qi, Xuan Wang, Jing Shen, Yafei Chi, Tiantian Li, Michelle M. Dunk, Yu An, Hongmei Huang, Kang Yu, Weili Xu, Rong Xiao, Yuandi Xi

**Affiliations:** ^1^ Beijing Key Laboratory of environment and aging, School of Public Health Capital Medical University Beijing 100069 China; ^2^ Department of Geriatrics Beijing Jishuitan Hospital Capital Medical University Beijing 102208 China; ^3^ School of Radiology Shandong First Medical University & Shandong Academy of Medical Sciences Taian 271016 China; ^4^ Department of Neurology and Department of Clinical Trial Center Beijing Tiantan Hospital Capital Medical University Beijing 100071 China; ^5^ China National Clinical Research Center for Neurological Diseases Beijing 100050 China; ^6^ Department of Experimental Animals Capital Medical University Beijing 100069 China; ^7^ Aging Research Center Department of Neurobiology Care Sciences and Society Karolinska Institutet Stockholm 17165 Sweden; ^8^ Medical Research Center Beijing Institute of Respiratory Medicine and Beijing Chao‐Yang Hospital Capital Medical University Beijing 100020 China; ^9^ Department of Clinical Nutrition Beijing Children's Hospital Capital Medical University National Center for Children's Health Beijing 100045 China; ^10^ Department of Clinical Nutrition and Department of Health Medicine Peking Union Medical College Hospital Beijing 100730 China

**Keywords:** FNDC5, irisin, lecithin, mild cognitive impairment, sarcopenia

## Abstract

Identifying the mechanistic targets of crosstalk between sarcopenia (SA) and mild cognitive impairment (MCI) is critical for screening high‐risk populations and exploring effective prevention and treatment strategies. In a nationwide multicenter prospective cohort study combined with an RCT study, it is found that indexes of muscle health reveal a strong predictive relationship with cognitive performance assessed using the Montreal Cognitive Assessment (MoCA). Furthermore, Random Forest models suggest that lecithin can predict both diseases. Erythrocyte lipid analysis and RCT study indicate the protective function of lecithin and the potential involvement of irisin in that process. In rodent models, phosphocholine (PC) alleviates learning and memory impairments and muscle attenuation in SAMP8 mice, while FNDC5/irisin knockdown accelerates brain and muscle damage or eliminates the protective effects of PC. Transcriptome analysis shows that PGC1α (the regulator of FNDC5) is regulated by PC treatment, and the results of knocking out PGC1α and FNDC5/irisin are consistent. Here it is found that muscle‐secreted FNDC5/irisin is a key target of “muscle‐brain” crosstalk, and lecithin may postpone the progression of MCI and SA by stimulating PGC1α‐FNDC5/irisin‐mediated cross‐protection of cognition and skeletal muscle.

## Graphical abstract

This study opens a new avenue for safeguarding cognition and muscle health, averting disability in older age, and treating age‐related pathologies through lecithin supplementation. It serves as a promising nonpharmacological intervention for the crosstalk of muscle and cognition. The protective effects of lecithin demonstrate in the investigations of both humans and mice provide strong support for the development of PC interventions.

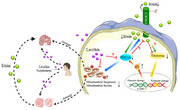

## 1 Introduction

Susceptibility to age‐related degenerative disease is an inevitable phenomenon in the human aging process and is commonly predicted by physiological dysfunction.^[^ ^1^ ^]^ One area of concern is cognitive dysfunction, which refers to deficits in learning/memory and impaired ability to perform daily activities such as conversing with others, reading, etc.^[^ ^2^, ^3^ ^]^ Physical performance, which denotes the capability to integrate and convert physiological stimuli into muscular actions, is another important aspect of daily executive functions, examples of which include grip strength, pace, and gait.^[^ ^4^, ^5^ ^]^ Both cognitive loss and physical dysfunction are key factors leading to injury, disability, and death, which has motivated the World Health Organization (WHO) to encourage the preservation of these functions for maintaining quality of life in older age.^[^ ^6^ ^]^

Mild cognitive impairment (MCI) is an early stage of dementia, of which the most common cause is Alzheimer's disease (AD). The conversion rate from MCI to AD is ≈10–15%.^[^ ^7^ ^]^ MCI is considered a window of opportunity for intervention to protect against further cognitive decline and the development of dementia.^[^ ^7^, ^8^, ^9^ ^]^ One potentially modifiable risk factor for MCI is sarcopenia (SA),^[^ ^10^ ^]^ which refers to the decline of skeletal muscle mass and function and can result in a progressive loss of physical function.^[^ ^11^ ^]^ These two prevalent degenerative diseases of aging are highly related to loss of intelligence and mobility, and there is evidence not only that individuals with SA have a higher risk of MCI, but also that those with MCI have a higher incidence of SA.^[^ ^10^, ^12^ ^]^ This phenomenon of crosstalk between sarcopenia and MCI provides a valuable opportunity for the early identification of at‐risk individuals. Further investigation of their relationship is critical to identify the overlapping mechanisms that might be key for developing therapeutic targets.

One prospective mechanistic target linking sarcopenia with MCI is irisin, a newly identified myokine with a variety of physiological regulatory effects that is secreted from muscle as a consequence of muscle contraction during shivering.^[^ ^13^ ^]^ Irisin is cleaved from fibronectin type III domain‐containing protein 5 (FNDC5) mediated by PGC1α.^[^ ^13^ ^]^ Irisin is able to cross the blood‐brain barrier with the help of peripheral transport before entering the central nervous system and inducing brain‐derived neurotrophic factor (BDNF) expression.^[^ ^14^ ^]^ Evidence also indicates that FNDC5/irisin rescues synaptic plasticity and memory impairments in AD mice.^[^ ^15^ ^]^ All of these provide strong evidence that FNDC5/irisin may serve as a bridge of crosstalk between the skeletal muscle system and the central nervous system.

Nutrition is an important modifiable behavior considered to be one of the main strategies to promote healthy aging and prevent age‐related diseases.^[^ ^16^ ^]^ Lecithin, also known as phosphatidylcholine (PC), is involved in multiple biological functions, such as maintaining the structural integrity and stability of cell membranes, supporting mitochondrial function, acting as a choline donor, and facilitating lipid transport and metabolism.^[^ ^17^, ^18^ ^]^ It is essential for both neurons and myocytes.

Postmortem brain sample studies revealed that reduced levels of PC can be found in the cerebral cortex of AD patients as compared to age‐matched controls and patients with Down syndrome, Parkinson's disease, and Huntington's disease.^[^ ^19^ ^]^ Dai et al. reported that all PCs were decreased in both the gray and white matter of AD brains, with a particularly significant reduction in PC(18:0/22:6) in the gray matter.^[^ ^20^ ^]^ Furthermore, a 23% reduction in total PC concentration and a 60% reduction in DHA‐enriched PC were observed in the erythrocytes of AD patients compared to control individuals.^[^ ^21^ ^]^ A cohort study conducted in Eastern Finland, involving 2497 men aged 42–60 years, found that each 50 mg day^−1^ higher lecithin intake was associated with a 10% lower risk of dementia.^[^ ^22^ ^]^ A double‐blind randomized controlled trial (RCT) in elderly Japanese individuals provides credible evidence that supplementation with soy lecithin significantly improved delayed word recall in those with low neuropsychological test scores, suggesting a beneficial effect of lecithin on memory in the early stages of dementia.^[^ ^23^ ^]^ Our previous research also revealed that PC could protect cerebrovascular endothelial cells from damage induced by Aβ25‐35,^[^ ^24^, ^25^ ^]^ which could be confirmed by PC preventing cerebrovascular injury induced by Aβ1‐40.^[^ ^26^, ^27^ ^]^

Although evidence for the benefits of lecithin on muscle health is limited, an important study completed by Russian researchers demonstrated that injection of PC could significantly increase the flounder muscle mass of rats, which had atrophied due to long‐term gravitational disuse.^[^ ^28^ ^]^ Interestingly, a study of Korean adolescents found a link between the composition of plasma phospholipid fatty acids and blood irisin levels.^[^ ^29^ ^]^ Moreover, our human lecithin RCT observed that lecithin could both improve MoCA scores and muscle indexes. Additionally, transcriptome results of PC‐administered mice suggest that PGC1α is an important gene target regulated by PC intervention. The research has shown that lecithin can efficiently hinder d‐galactosamine (d‐GalN)‐induced liver damage in rats, reduce the expression of p53 mRNA in liver cells, and maintain the stability of the mitochondrial membrane potential (MMP). Meanwhile, p53 and PGC1α play crucial roles in regulating the telomere‐mitochondrial axis.^[^ ^30^ ^]^

Based on these prior reports, we propose a hypothesis that lecithin may exert cross‐protective effects on cognition and muscle health through the regulation of the PGC1α‐FNDC5/irisin signaling pathway. In the present study, we performed a 24‐week RCT of older adults aged 65 years or above from Chinese cohorts (the Effect of Dietary Nutrition on the Cognitive Function and Sarcopenia in middle‐aged and elderly people [EDNCS] cohort) to evaluate the effects of PC on cognitive function and muscle health. Then, we examined possible contributing mechanisms in a complementary study of 9‐month‐old SAMP8 mice, SAMR1 mice, APP/PSI mice, and C57BL/6J mice, in which learning memory capacity, assessment of sarcopenia, and cerebrovascular function tests were examined following 8 weeks of PC administration.

## 2 Results

### 2.1 Variable Importance Plots for Predicting MOCA, SMM, SMI, and FFMI in Participants

**Figure 1:**
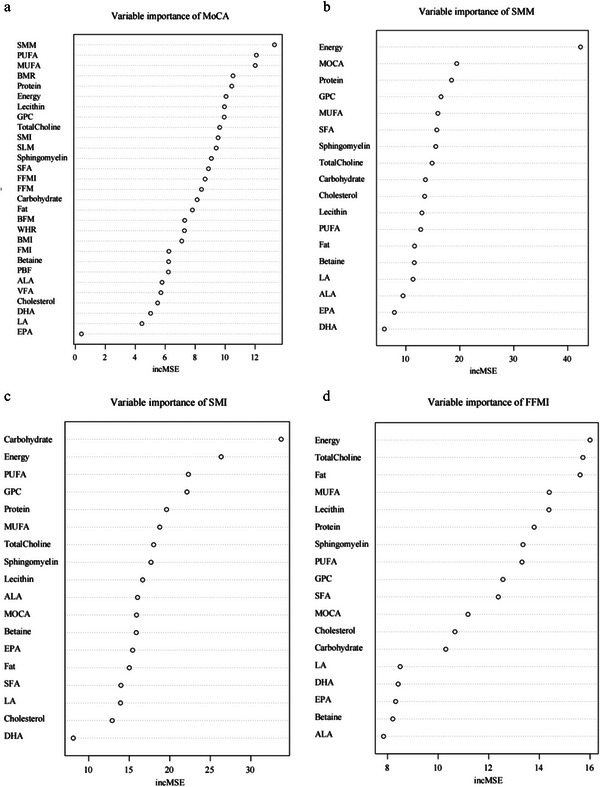
Variable importance plots for different features. a,b) Variable importance plots of the random forest model in the prediction of MoCA a), SMM b), SMI c), and FFMI d). An increase in MSE (incMSE) is used to evaluate the importance of each variable. The incMSE represents the increase in MSE when a variable changes. If a variable is important, the incMSE will be large when it changes, and vice versa.

Variable importance plots for different features in the prediction of MoCA, skeletal muscle mass (SMM), skeletal muscle index (SMI), and the fat‐free mass index (FFMI) are provided in **Figure** **1**a–d. First, the contribution rankings of SMM, lecithin, and SMI in the prediction of MoCA are within the top ten (Figure 1a). In addition, lecithin and SMM show positive overall trends with MoCA (Figure S1, Supporting Information). Second, the contribution of MOCA to SMM is second only to energy (Figure 1b). Notably, in the second and third follow‐up data, the prediction accuracy remains high, further supporting the positive relationship with SMM (Figure S2, Supporting Information). Lecithin ranks eleventh in its contribution to predicting SMM, preceded by protein, glycerophosphocholine, monounsaturated fatty acid (MUFA), saturated fatty acid (SFA), sphingomyelin, total choline, and carbohydrate (Figure 1b). In addition, in terms of the contribution of lecithin and MOCA to SMI prediction, the former ranks ninth while the latter ranks eleventh (Figure 1c). Both indicators demonstrate reliable predictive capabilities with respect to SMI and evidence positive overall associations (Figure S3, Supporting Information). Similarly to SMI, for FFMI, the contribution of lecithin ranks fifth, while that of MOCA ranks eleventh (Figure 1d). Both indicators display noteworthy prediction accuracy for FFMI and positive overall trends (Figure S4, Supporting Information).

### 2.2 PC with Unsaturated Fatty Acid in Human Erythrocyte Played a Key Role in MCI Combined with SA

In view of the dual important roles of dietary lecithin and its metabolite choline on cognition and skeletal muscle health in the cohort prediction model, targeted lipidomic analysis was conducted by UHPLC‐QTRAP6500+‐MS/MS for testing the erythrocyte of 15 samples in each group (Figure S5, Supporting Information). Compared to the control group, the levels of PC (18:2/20:4) containing more polyunsaturated fatty acid (PUFA) were significantly reduced in the MCI combined with the SA group. Whereas, PC (18:0/18:0), PC (18:0/18:2), and PC (18:0/18:1), which contain more SFA, had an increasing trend in the SA group. Correspondingly, we calculated the percentage of PC of specific fatty acids such as 16:0, 18:0, 20:3, 20:4, etc. to the total expression of PC. The percentage of PC containing PUFA of 20:4 or 20:3 was significantly lower in MCI combined with the SA group than that in the control group, while the percentage of PC containing SFA of 16:0 or 18:0 had no differences.

### 2.3 Lecithin Administration Improved Cognitive States and Increased Irisin Levels in the Elderly

**Figure 2:**
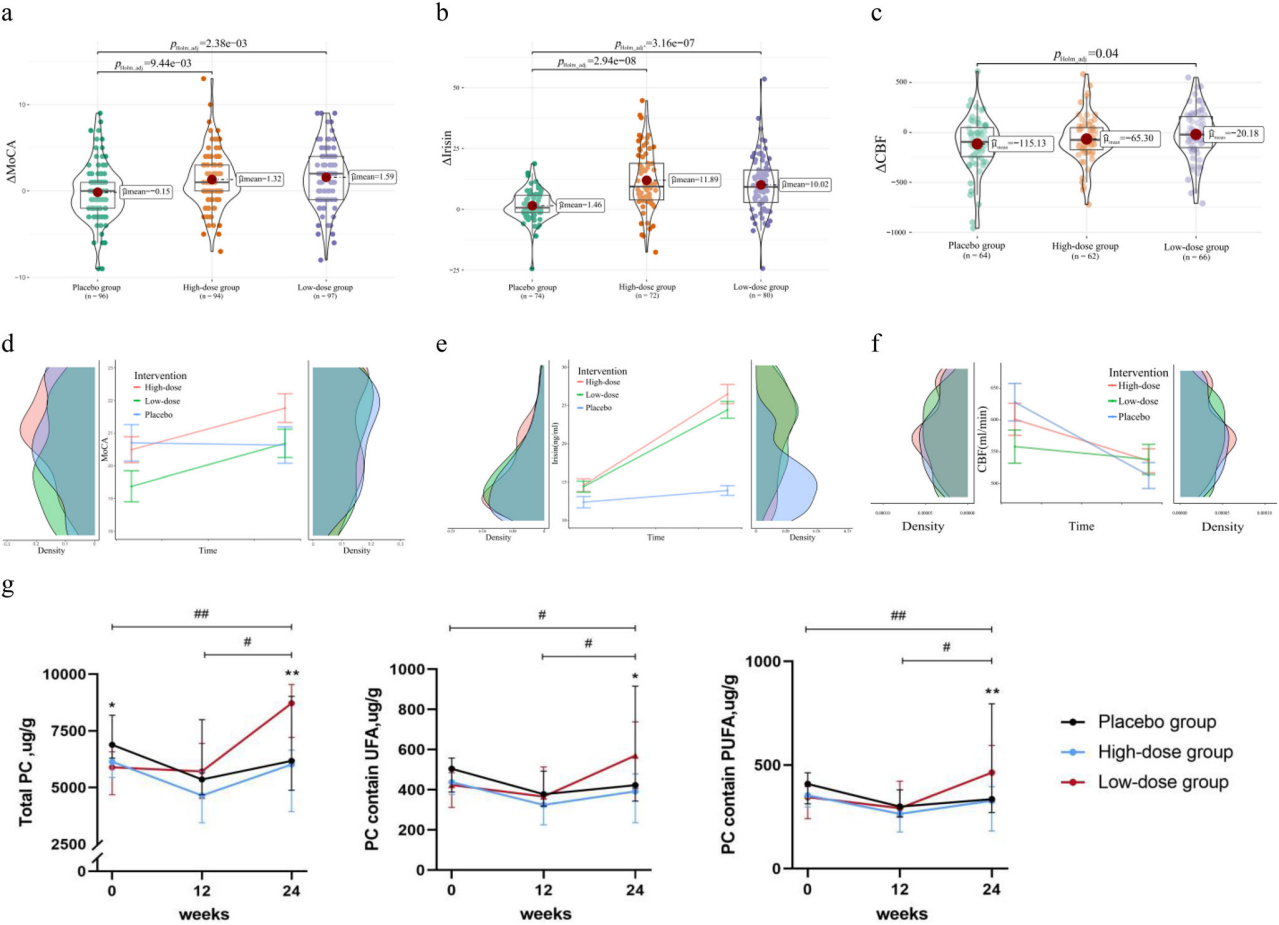
Intervention results. a) Comparisons of ΔMoCA pre‐ and post‐intervention among palcebo, *n* = 96, high‐dose, *n* = 94 and low‐dose groups, *n* = 97. b) Comparisons of Δirisin pre‐ and post‐intervention among palcebo, *n* = 74, high‐dose, *n* = 72 and low‐dose groups, *n* = 80. c) Comparisons of ΔCBF pre‐ and post‐intervention among palcebo, *n* = 64, high‐dose, *n* = 62 and low‐dose groups, *n* = 66. d,f) Line and density plot of MoCA (d), irisin (e), and CBF (f) levels pre‐ and post‐intervention among placebo, high‐dose, and low‐dose groups. Errors indicate the standard error of the mean. The left density plot represents conditions pre‐intervention, right density plot represents conditions post‐intervention. g) Targeted lipidomics were performed to test total PC, PC containing unsaturated fatty acids, and PC containing polyunsaturated fatty acids among Placebo, High‐dose, and Low‐dose groups at pre‐, mid‐, and post‐intervention. *n*  =  15 per group. Data presented as mean ± s.e.m, median ± se, 25th and 75th percentiles. Welch's ANOVA, One‐way ANOVA, Kruskal–Wallis tests, two‐tailed Student's *t*‐tests, and Mann–Whitney U tests were performed. ^#^, *p* < 0.01 in low dose group, ^##^, *p* < 0.001 in low dose group, ^*^ *p* < 0.05 among the three groups, ^**^ *p* < 0.01 among the three groups.

In this study, we found that the three intervention groups differed significantly in terms of delta (Δ) MoCA (F = 8.17, P = 3.92 × 10–4) (**Figure** **2**a). Specifically, the high‐dose and low‐dose groups showed some improvement in MoCA scores, while the placebo group experienced a slight decrease. Similarly, there was a significant difference in Δ irisin among the three intervention groups (F = 30.93, P = 8.66 × 10–12). Notably, the low‐dose and high‐dose groups led to a significant increase in irisin concentration, while the placebo group did not (Figure 2b). The Δ cerebral blood flow (CBF) showed no significant difference among the three intervention groups (F = 1.141, P = 0.286). In terms of CBF, although there was no significant difference in ΔCBF among the three groups, the low‐dose group showed significant ΔCBF increase compared to placebo (*p* = 0.04), and the high‐dose group also demonstrated a slight increase in ΔCBF (Figure 2c). Although SMI did not differ significantly among the different intervention groups, it is worth noting that the degeneration trend of SMM, SMI, and soft lean mass (SLM) was relieved in the high‐dose group (Figure S6, Supporting Information).

In order to further validate the effectiveness of PC treatment, we conducted targeted lipidomics analysis in all three groups (placebo, low, and high PC) at baseline, mid‐treatment period (12 weeks, if blood samples were available), and end‐treatment period (at 24 weeks). The ideal increase in total PC, PC containing polyunsaturated fatty acid, and PC containing unsaturated fatty acid of erythrocyte could be found both in the mid‐treatment period and end‐treatment period, especially in the low‐dose group (Figure 2g).

### 2.4 Learning and Memory Impairment Combined with Muscle Attenuation in SAMP8 Mice and the Mechanism of FNDC5/Irisin

#### 2.4.1 SAMP8 Mice Showed Learning and Memory Impairment Combined with Muscle Attenuation

We first performed a comprehensive characterization of the motor and cognitive abilities of male SAMP8 mice and APP/PS1 mice. In our study, SAMP8 mice exhibited suboptimal performance in all tests assessing cognitive abilities and muscle function. However, the APP/PS1 mice only showed cognitive impairments (**Figure** **3**a–i).

Figure 3SAMP8 mice show aging‐related learning memory impairment and muscle decay. a) latency to reach the target platform of MWM. *n*  =  6 per group. b,c) DI (b) of NOR and exploration time for familiar (F) and new objects (N) (c). SAMR1, *n* = 7, SAMP8, *n* = 8, WT, *n* = 8, APP∖PS1, *n* = 6. d,e) Grip strength of limbs (d) and forelimb (e). f) Hanging grid test. g) Rotarod. h) Weights of gastrocnemius muscles. i) lean mass. *n*  =  6 per group. j,k) Hippocampus (j) and whole brain (k) of CBF. l–o) PWV (l,m) and blood flow velocity (n,o) in both carotid arteries. *n*  =  6 per group. p–s) Protein levels (p,q) and mRNA levels (r,s) of FNDC5/irisin in the hippocampus. t–w) Protein levels (t,u) and mRNA levels (v, w) of FNDC5/irisin in gastrocnemius. *n* = 6 independent tissue donors for mRNA; 3 donors for protein levels. x) Plasma irisin levels. *n*  =  6 per group. Data in all line or bar graphs are shown as mean ± s.e.m. For the box‐and whiskers‐graphs, minima, maxima, median, 25th and 75th percentiles are shown, with whiskers indicating the smallest and largest values. Two‐tailed Student's *t*‐tests, Welch's *t*‐tests, and Mann–Whitney U tests were performed. ^*^ *p* < 0.05; ^**^ *p* < 0.01.

**Figure:**
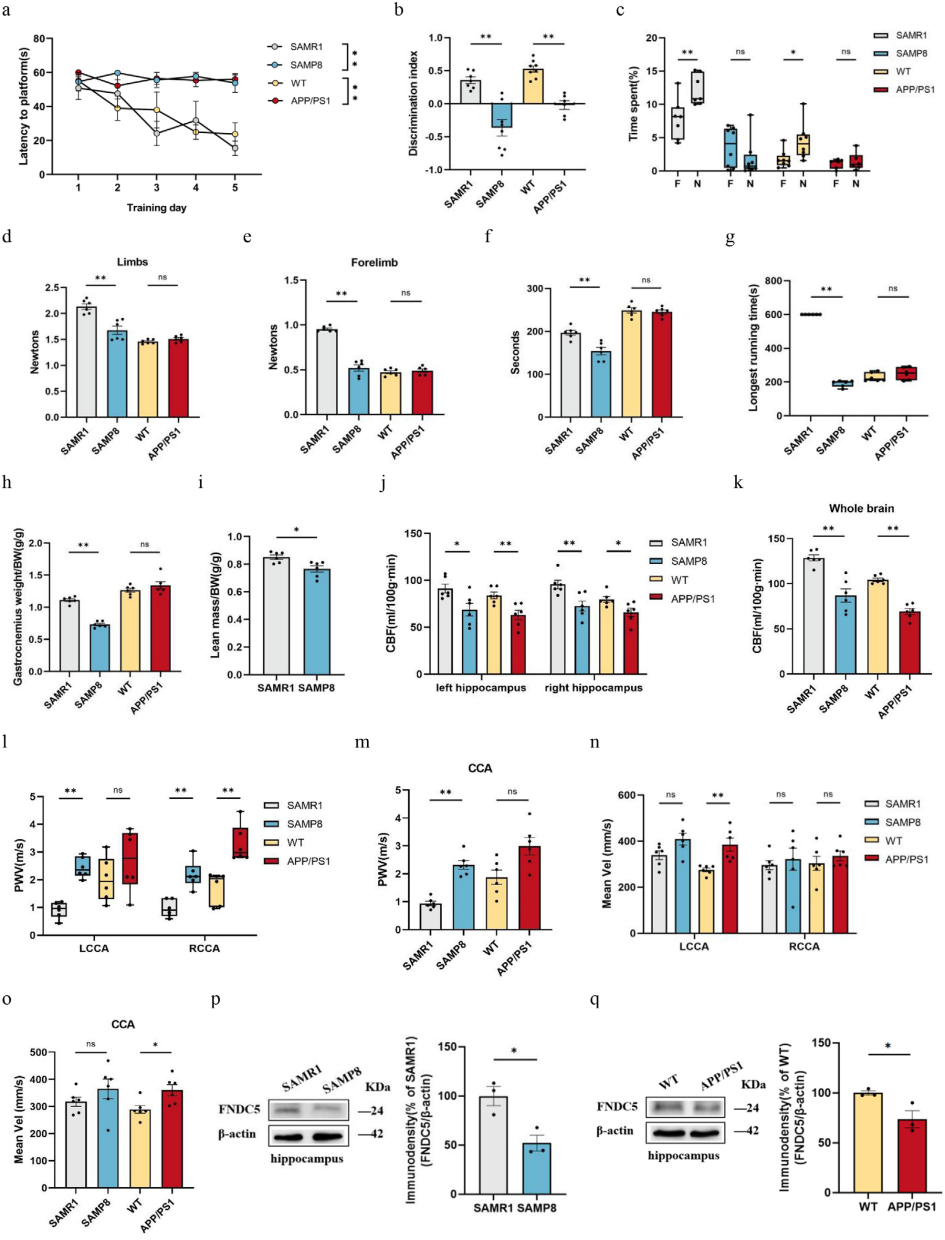

**Figure:**
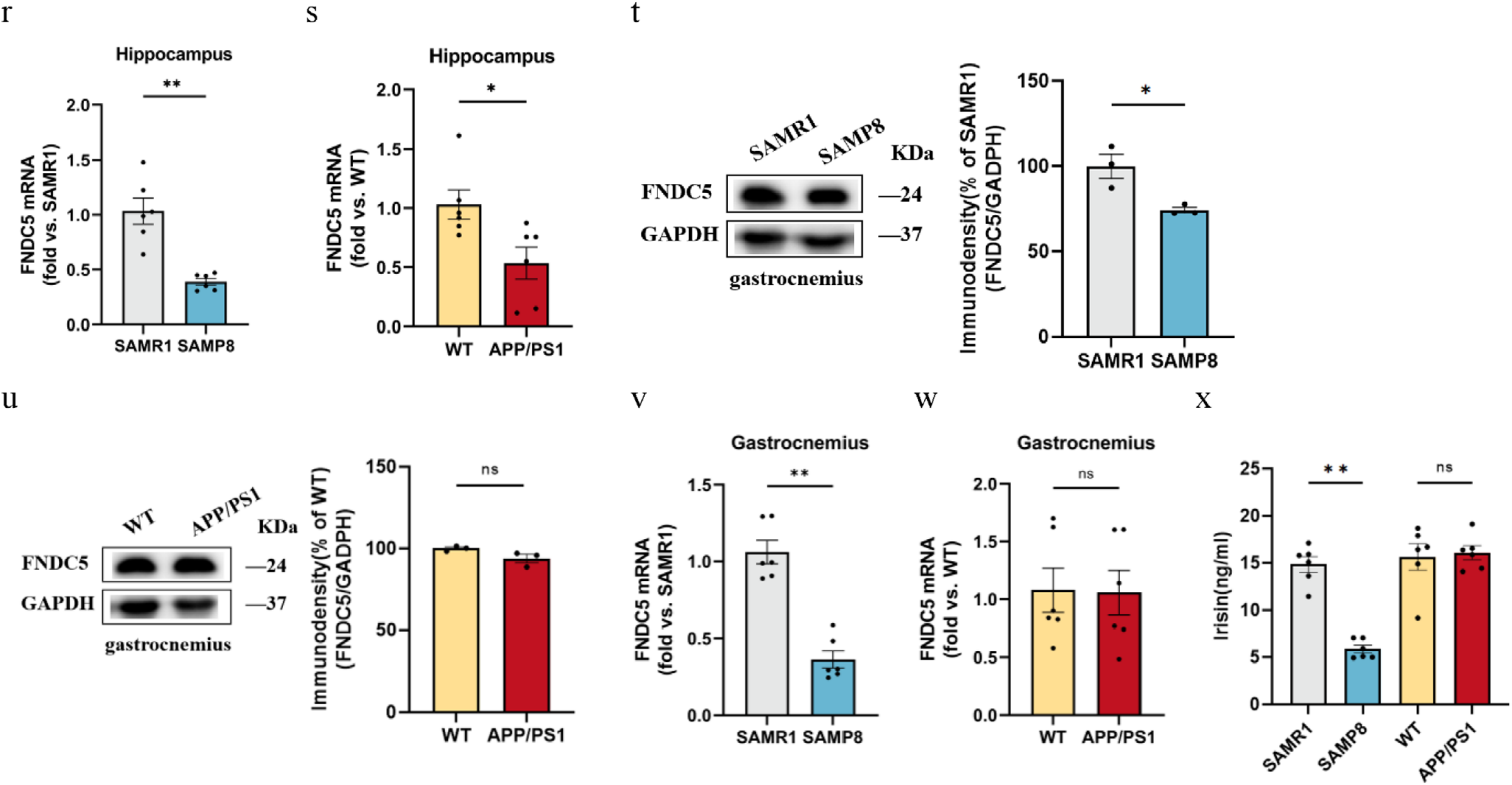

#### 2.4.2 Cerebral Vascular Injury of SAMP8 Mice

CBF is an important way for transporting peripheral irisin into the brain. In that, we used arterial spin‐labeled MRI (ASL‐MRI) and Doppler ultrasound to detect CBF and vascular function. Results showed that the average CBF of the whole brain and hippocampus in SAMP8 and APP/PS1 mice was lower than in SAMR1 and WT mice (Figure 3j,k). Furthermore, the Doppler ultrasound examination revealed a marked elevation in pulse wave velocity (PWV) and carotid artery blood flow velocity in both SAMP8 and APP/PS1 mice (Figure 3l–o).

#### 2.4.3 FNDC5/Irisin was Downregulated in SAMP8 Mice

In line with the discoveries of prior investigations, compared with SAMR1 mice and WT mice, SAMP8 mice and APP/PS1 mice showed significant decreases in the FNDC5/irisin protein levels and FNDC5 mRNA expression in the brain. Additionally, only SAMP8 mice experienced decreases in FNDC5/irisin levels in the muscle and circulation (Figure 3p–x).

#### 2.4.4 FNDC5/Irisin Knockdown Accelerated Brain and Muscle Damage in SAMP8 Mice

Adeno‐associated virus (AAV)‐FNDC5‐shRNAs was used to knockdown FNDC5 in the hippocampus of SAMP8 mice (Figure S7a,b, Supporting Information). This resulted in aggravated impairment of memory in the Morris Water Maze (MWM) and Novel Object Recognition (NOR) task, along with reduced muscle performance such as grip strength, wire suspension test, rotarod test, and even a decrease of gastrocnemius weight/BW and lean mass/BW (Figure S7c–k, Supporting Information). Meanwhile, according to vascular ultrasound, the absence of FNDC5/irisin appeared to accelerate cerebrovascular damage (Figure S7l–q, Supporting Information). In addition, hippocampus FNDC5/irisin knockdown led to a significant elevation of FNDC5 mRNA expression in the hindlimb muscles of mice, and also raised levels of FNDC5/irisin in peripheral plasma (Figure S7r–t, Supporting Information). However, the increased SA‐β‐Gal positive staining area in the hippocampus (Figure S7u, Supporting Information), as well as elevated levels of inflammation, could be found both in the brain and muscle after knockdown of hippocampus FNDC5/irisin (Figure S7v, Supporting Information).

#### 2.4.5 FNDC5/Irisin Overexpression Rescued Brain and Muscle Attenuation in SAMP8 Mice

We further investigated the effects of FNDC5/irisin reintroduction on brain and muscle impairments. SAMP8 mice were infected via i.c.v. with a control vector (AAV‐GFP) or with AAV‐FNDC5 (Figure S8a,b, Supporting Information). As expected, boosting brain FNDC5/irisin levels rescued all defects in behavioral experiments and related indexes of brain and muscle, in addition to reducing senescence and some inflammatory factors in SAMP8 (Figure S8, Supporting Information).

### 2.5 The Critical Role of PC with Unsaturated Fatty Acid on Erythrocyte in SAMP8 Mice

Targeted lipidomic was also used to test the erythrocyte PC expressions in SAMP8 mice. Compared with the mice of SAMR1, PC (18:1/20:1) and PC (18:1/20:3) which contained more unsaturated fatty acids were downregulated in SAMP8 mice. However, PC (16:0/18:0), PC (16:0/14:0), PC (18:0/16:1), PC (18:0/18:2), PC (16:0/18:2), and PC (14:0/18:1) which contained more saturated fatty acids were upregulated in SAMP8 (Figure S9, Supporting Information).

### 2.6 PC Treatment Prevented Brain and Muscle Impairment and Regulated PC Components of Erythrocyte in SAMP8 Mice

PC treatment alleviated the impaired performance of SAMP8 mice in MWM and NOR tests (**Figure** **4**a–c), while also improving grip strength, wire suspension test, rotarod test, gastrocnemius weight/BW, and lean mass/BW (Figure 4d–i). Additionally, the PC intervention also had a significant impact on vascular function. CBF was significantly higher in the PC 200 group compared to the model group (Figure 4j,k), while the PWV was lower in the PC 200 group, indicating an obvious improvement in cerebrovascular function (Figure 4l–o). Moreover, PC administration upregulated FNDC5/irisin mRNA and protein expression in the hippocampus, gastrocnemius, and plasma of SAMP8 mice (Figure 4p–t). Correspondingly, SA‐β‐gal staining in the hippocampus, the level of TNF‐α in the brain and gastrocnemius, as well as IL‐7 and ICAM‐1 in gastrocnemius were all down‐regulated by treatment of 200mg kg^−1^ PC (Figure 4u,v).

Figure 4Administration of PC alleviates learning memory impairment and muscle decay. a) latency to reach the target platform of MWM. *n*  =  6 per group. b,c) DI (b) of NOR and exploration time for familiar (F) and new objects (N) (c). Ctrl, *n* = 8, Model, *n* = 8, PC200, *n* = 7, PC100, *n* = 8. d,e) Grip strength of limbs (d) and forelimb (e). f) Hanging grid test. g) Rotarod. h) Weights of gastrocnemius muscles. i) lean mass. *n*  =  6 per group. j,k) Hippocampus (j) and whole brain (k) of CBF. l–o) PWV (l,m) and blood flow velocity (n,o) in both carotid arteries. *n*  =  6 per group. p,q) Protein levels (p) and mRNA levels (q) of FNDC5/irisin in the hippocampus. r,s) Protein levels (r) and mRNA levels (s) of FNDC5/irisin in gastrocnemius. *n* = 6 independent tissue donors for mRNA; 3 donors for protein levels. t) Plasma irisin levels. u) Representative images and quantitative analysis of SA‐β‐gal staining of hippocampus (scale bar = 200 µm; *n*  =  3). v) Inflammation levels in the hippocampus and muscles. *n*  =  4 per group. w) Comparison of PCs containing different fatty acids in each group. Ctrl, *n* = 6, Model, *n* = 8, PC200, *n* = 6. Data in all line or bar graphs are shown as mean ± s.e.m. For the box‐and whiskers‐graphs, minima, maxima, median, 25th and 75th percentiles are shown, with whiskers indicating the smallest and largest values. Two‐tailed Student's *t*‐tests, Welch's *t*‐tests, ANOVA, and Kruskal–Wallis tests were performed. ^*^ *p* < 0.05; ^**^ *p* < 0.01.

**Figure:**
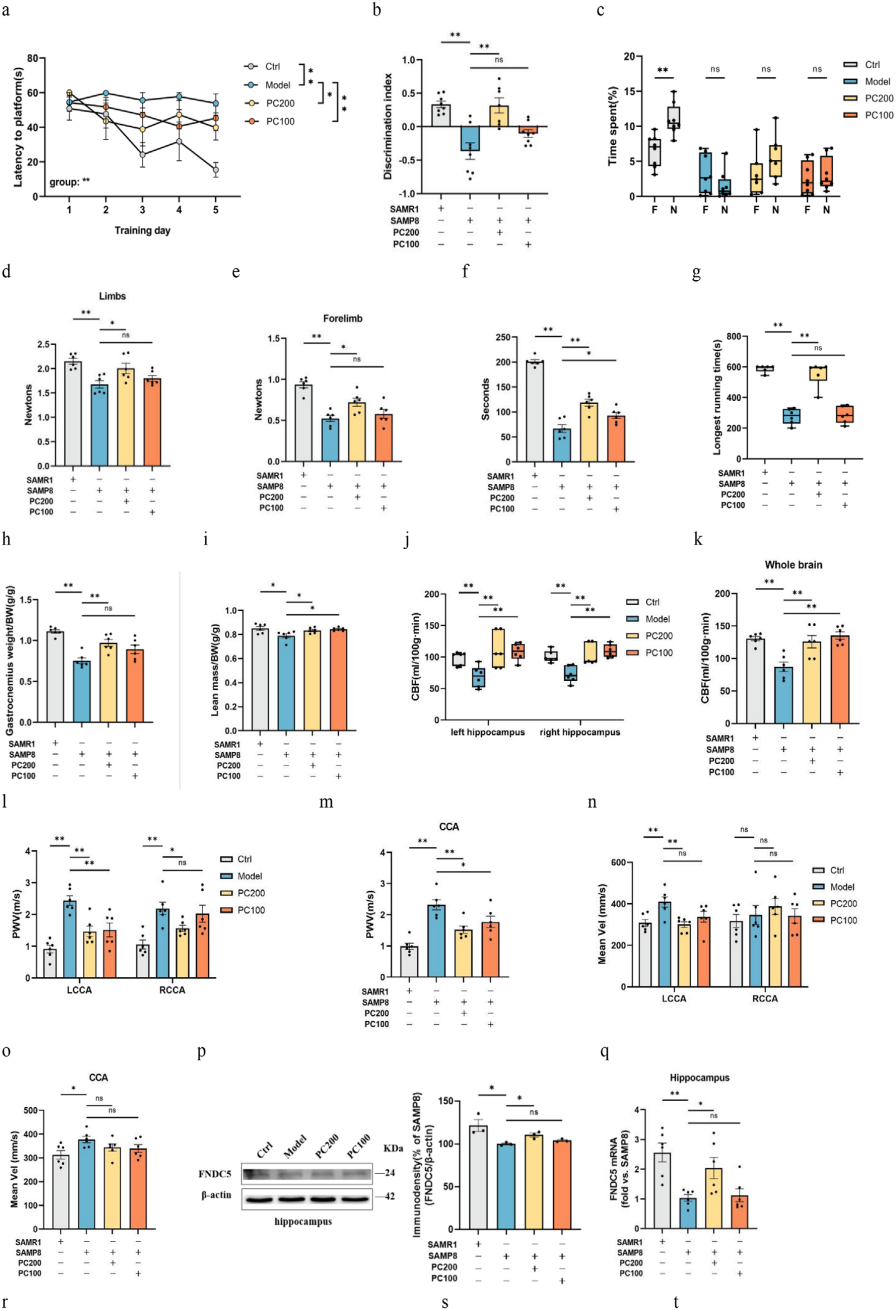

**Figure:**
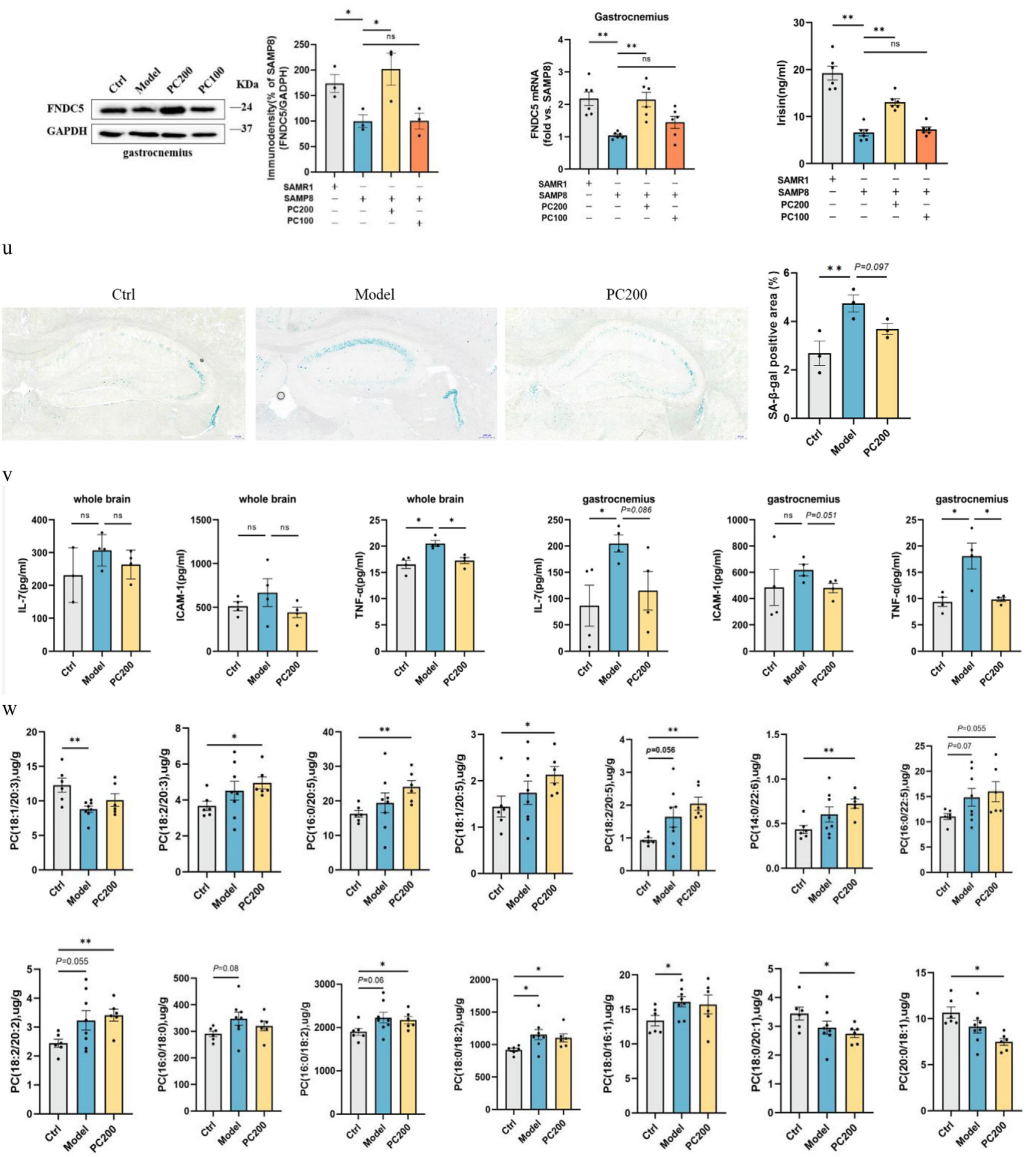

Targeted lipidomic analysis showed PC administration could lead to an up‐regulation of PCs rich in unsaturated fatty acids, such as PC (18:1/20:3), PC (18:2/20:3), PC (18:1/20:5), PC (18:2/20:5), and PC (18:2/20:2). However, PC (16:0/18:0), PC (16:0/18:2), and PC (18:0/18:2), which are rich in saturated fatty acids, were down‐regulated by PC treatment (Figure 4w). Apart from the above data, novel results showed that supplementation of PC could significantly increase the expression of PC‐rich Eicosapentaenoic Acid (EPA, C20:5) and Dihomo‐γ‐Linolenic Acid (DGLA, C20:3) on erythrocyte (Table S1, Supporting Information).

### 2.7 PGC1α‐FNDC5/Irisin is the Key Mechanism of PC Mediating Brain and Muscle Crosstalk in SAMP8 Mice

#### 2.7.1 Transcriptomic Changes Resulting from PC Treatment in SAMP8 Mice

We isolated total RNA from the hippocampus of the model and PC200 groups and performed RNA‐seq analysis. We identified 1634 genes that were significantly differentially expressed in the hippocampus of the model group versus the PC200 group (Figure S10a, Supporting Information). Notably, the PC200 group exhibited significant up‐regulation in the expression of PGC1α (Ppargc1a, the regulator of FNDC5) in comparison to the model group (log2FoldChange = 0.32, *p*‐value = 0.045). Gene ontology (GO) enrichment analysis on all significantly upregulated gene sets in PC200 mice revealed an over‐representation of GO components such as regulation of neurotransmitter levels, regulation of transmembrane transport, learning or memory, cognition, lipid biosynthetic process, muscle system process, and mitochondrion organization (Figure S10b, Supporting Information). In addition, GO components related to immune inflammation such as regulation of the immune effector process, positive regulation of the immune effector process, regulation of defense response, and positive regulation of cytokine production were down‐regulated in PC200 mice (Figure S10c, Supporting Information). In addition to the GO enrichment assessment, we performed Kegg (Kyoto Encyclopedia of Genes and Genomes) pathway analysis on significantly upregulated genes and down‐regulated genes of the hippocampus of the PC200 group. Most interestingly, significantly upregulated pathways included the phosphatidylinositol signaling system, pathways of neurodegeneration–multiple diseases, and Alzheimer's disease (Figure S10d, Supporting Information). Significantly down‐regulated pathways included the NOD‐like receptor signaling pathway, NF‐kappa B signaling pathway, lipid, and atherosclerosis (Figure S10e, Supporting Information). Kegg pathway analysis performed here strongly suggested that PC intervention in SAMP8 mice alters signaling pathways associated with neurodegenerative diseases and immune inflammation in the brain. Based on GO and KEGG analyses, a signal net of genes containing PGC1α (Ppargc1a) was identified (Figure S10f, Supporting Information).

#### 2.7.2 FNDC5/Irisin Deletion Abolished the Alleviating Effects of PC on Brain and Muscle Attenuation

To explore the possible role of FNDC5/irisin in PC treatment, we investigated whether the absence of FNDC5 would affect the protective effects of PC on learning and memory, as well as muscle performance in SAMP8 mice. The results showed that infusion of innocuous shRNA into mice followed by intragastric administration of PC resulted in an increase in FNDC5/irisin mRNA and protein expression in the hippocampus. However, in FNDC5 knockdown mice, the FNDC5/irisin promoting effect of PC on both mRNA and protein in the hippocampus was eliminated (**Figure** **5**a,b). It was noteworthy that the knockdown of FNDC5 in the brain did not block the upregulation of PC on FNDC5/irisin in muscle and plasma (Figure 5c–e).

Figure 5Deletion of FNDC5/irisin abrogates PC intervention‐induced protection against the brain and muscle of mice. a,b) Protein levels (a) and mRNA levels (b) of FNDC5/irisin in the hippocampus. c,d) Protein levels (c) and mRNA levels (d) of FNDC5/irisin in gastrocnemius. *n* = 6 independent tissue donors for mRNA; 3 donors for protein levels. e) Plasma irisin levels. f) latency to reach the target platform of MWM. *n*  =  6 per group. g,h) DI (g) of NOR and exploration time for familiar (F) and new objects (N) (h). R1‐GFP‐CMC, *n* = 7, P8‐GFP‐CMC, *n* = 6, P8‐GFP‐PC200, *n* = 6, P8‐shFNDC5‐CMC, *n* = 6, P8‐shFNDC5‐PC200, *n* = 6. i,j) Grip strength of limbs (i) and forelimb (j). k) Hanging grid test. l) Rotarod. m) Weights of gastrocnemius muscles. n) lean mass. *n*  =  6 per group. o,p) Hippocampus (o) and whole brain (p) of CBF. q–u) PWV (q,r) and blood flow velocity (s,t) in both carotid arteries. *n*  =  6 per group. Data in all line or bar graphs are shown as mean ± s.e.m. For the box‐and whiskers‐graphs, minima, maxima, median, 25th and 75th percentiles are shown, with whiskers indicating the smallest and largest values. Two‐tailed Student's *t*‐tests, Welch's *t*‐tests, ANOVA, and Kruskal–Wallis tests were performed. *p < 0.05; ^**^ *p* < 0.01.

**Figure:**
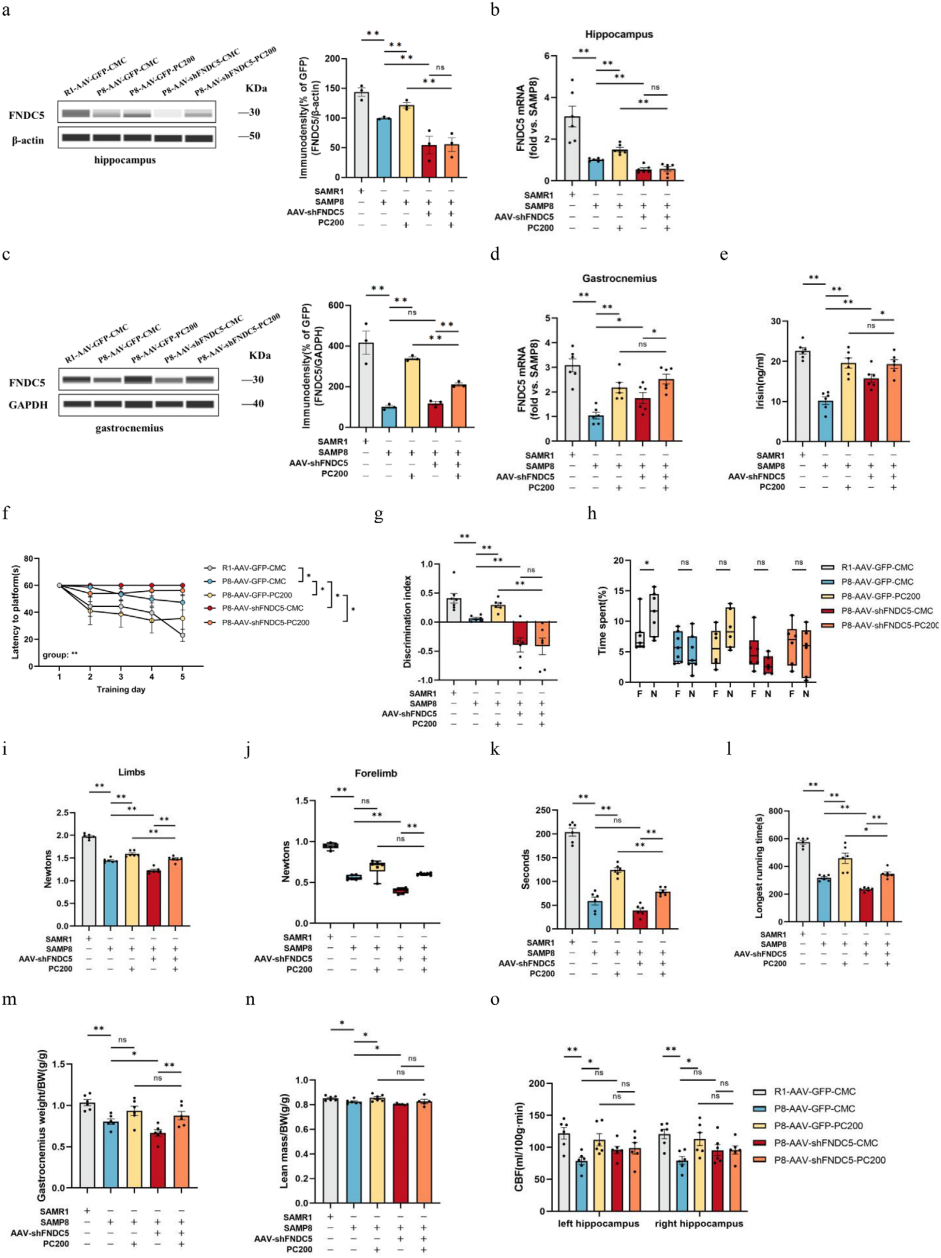

**Figure:**
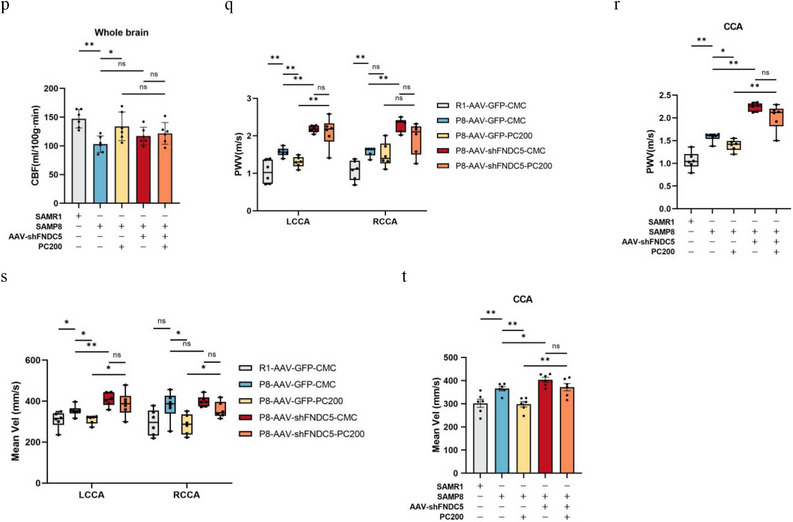

Accompanying the results of FNDC5/irisin, PC treatment did not rescue the capability of SAMP8 mice injected with AAV‐shFNDC5 on the latency of the MWM test and the DI of NOR task (Figure 5f–h). The same results were manifested in the evaluation tests for SA (Figure 5i–n). Furthermore, the cerebrovascular protecting effects of PC were alleviated by the knockdown of FNDC5 in the brain (Figure 5o–t).

Given that PGC1α is both the regulatory target of PC and the regulator of irisin secretion, the hippocampus PGC1α knockdown model of SAMP8 was also conducted by AAV‐shPGC1α in the present study (**Figure** **6**a,b). The results showed that silencing of PGC1α resulted in a reduction of FNDC5/irisin in the hippocampus (Figure 6c–f), but also feedback upregulation of PGC1α and FNDC5/irisin in the muscle (Figure 6g–j). As expected, PC administration did not reverse the downregulation of FNDC5/irisin in the hippocampus caused by knockdown of PGC1α (Figure 6e,f) but did lead to upregulation of PC on PGC1α and FNDC5/irisin in muscle (Figure 6g–j). This phenomenon contributed to all blockage of PC on improving the capability of learning and memory (Figure 6l–n), as well as the cerebrovascular function of SAMP8 (Figure 6o–t), but not in the muscle function and attenuation indexes (Figure 6u–z).

Figure 6Deletion of PGC1α abrogates PC intervention‐induced protection against the brain and muscle of mice. a,b) Levels of PGC1α mRNA (a) and FNDC5/irisin protein (b) in control (AAV‐GFP) compared to AAV‐shPGC1α‐injected mice. c–f) Protein levels (c,e) and mRNA levels (d,f) of PGC1α and FNDC5 in the hippocampus. g–j) Protein levels (g,i) and mRNA levels (h,j) of PGC1α and FNDC5 in gastrocnemius. *n* = 6 independent tissue donors for mRNA; 3 donors for protein levels. k) Plasma irisin levels. *n*  =  6 per group. l) latency to reach the target platform of MWM. *n*  =  6 per group. m,n) DI (m) of NOR and exploration time for familiar (F) and new objects (N) (n). R1‐GFP‐CMC, *n* = 7, P8‐GFP‐CMC, *n* = 6, P8‐GFP‐PC200, *n* = 6, P8‐shPGC1α‐CMC, *n* = 6, P8‐shPGC1α‐PC200, *n* = 6. o,p) Hippocampus (o) and whole brain (p) of CBF. q–t) PWV (q,r) and blood flow velocity (s,t) in both carotid arteries. *n*  =  6 per group. u,v) Grip strength of limbs (u) and forelimb (v). w) Hanging grid test. x) Rotarod. y) Weights of gastrocnemius muscles. z) lean mass. *n*  =  6 per group. Data in all line or bar graphs are shown as mean ± s.e.m. For the box‐and whiskers‐graphs, minima, maxima, median, 25th and 75th percentiles are shown, with whiskers indicating the smallest and largest values. Two‐tailed Student's *t*‐tests, Welch's *t*‐tests, ANOVA, and Kruskal–Wallis tests were performed. ^*^ *p* < 0.05; ^**^ *p* < 0.01.

**Figure:**
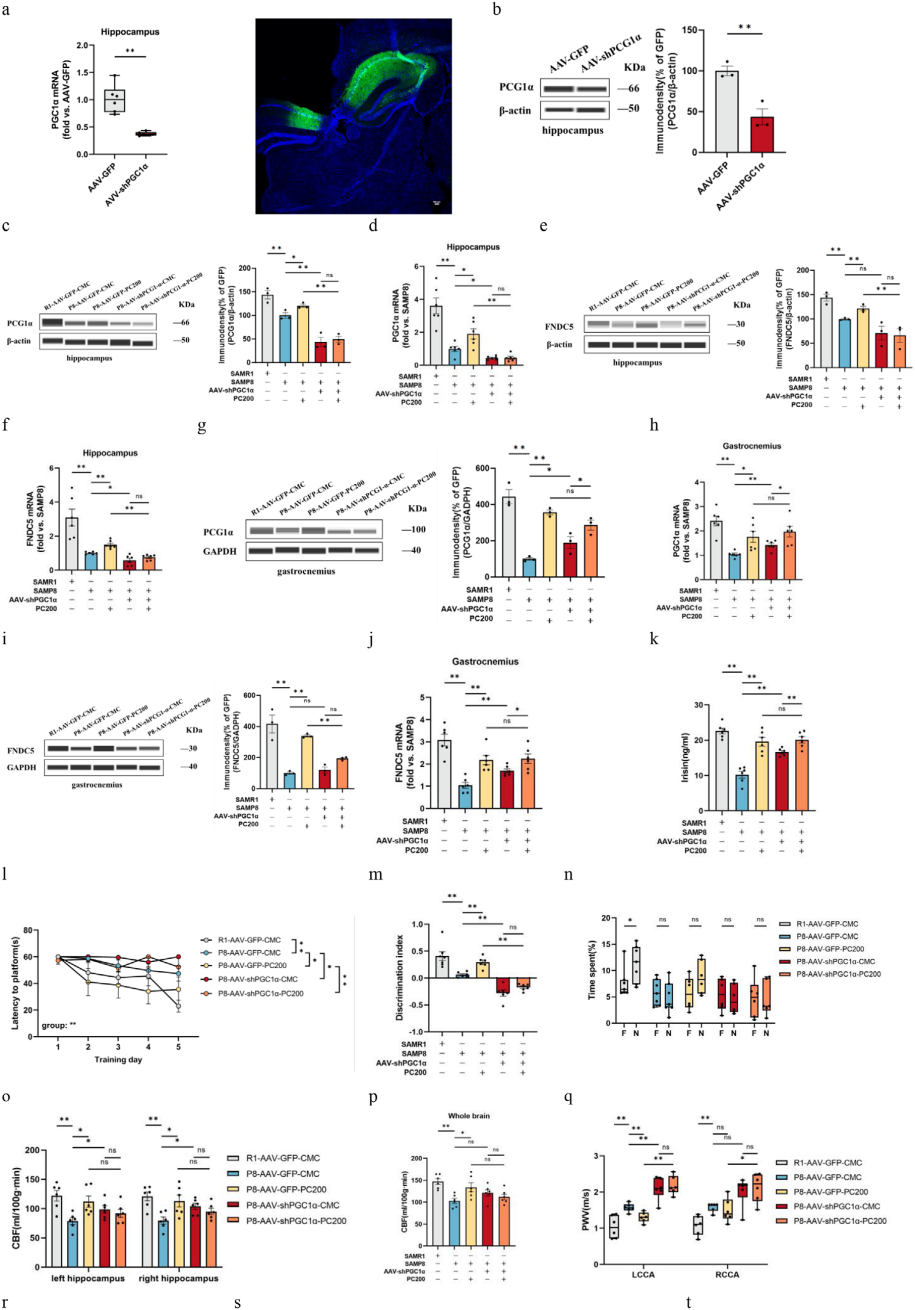

**Figure:**
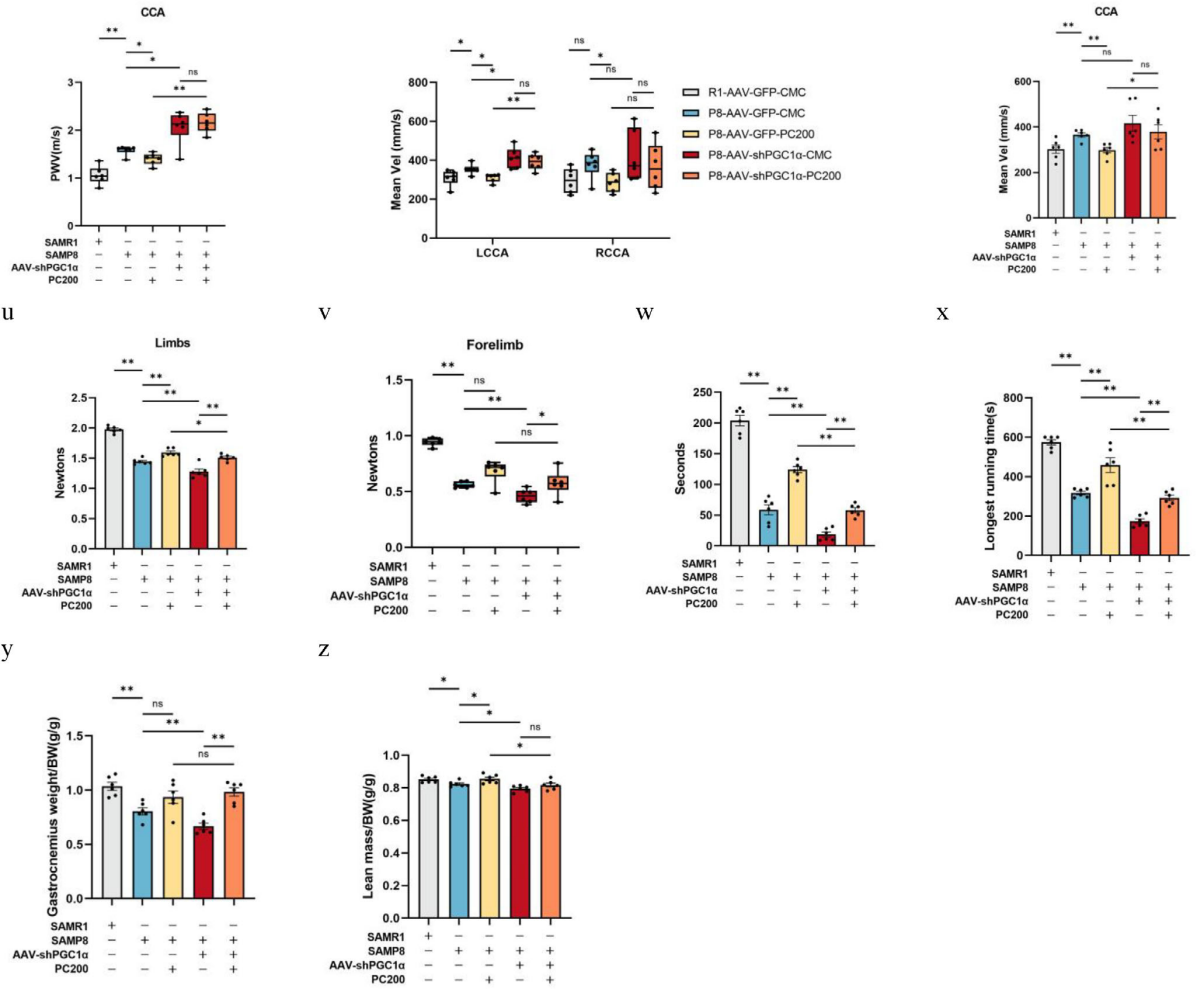

#### 2.7.3 Telomeres‐Mitochondria Protection of PC Through PGC1α‐FNDC5/Irisin Signaling Pathway

**Figure 7:**
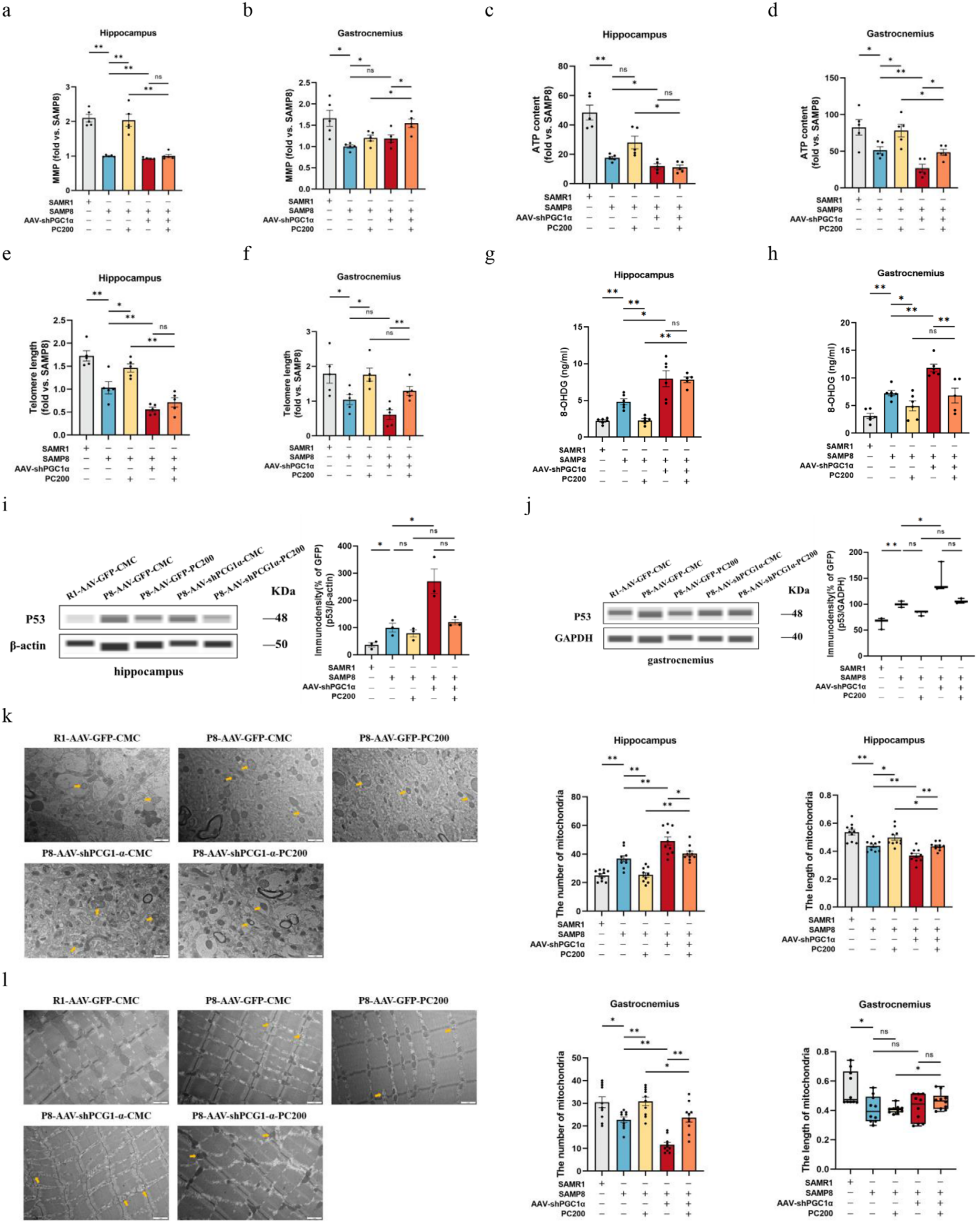
PGC1α deficiency impaired mitochondrial function and shortened telomere length in the hippocampus and muscle. a–j) MMP (a), ATP content (c), telomere length (e), protein level of 8‐OHDG (g), and P53 (i) in the hippocampus. MMP (b), ATP content (d), telomere length (f), the protein level of 8‐OHDG (h) and P53 (j) gastrocnemius. *n*  =  5 per group. k) Represents mitochondrial number and length in the hippocampus. Abnormal mitochondria (yellow arrows) from the hippocampus (scale bar = 2 µm; *n* = 10). l, Represents mitochondrial number and length in gastrocnemius. Abnormal mitochondria (yellow arrows) from the gastrocnemius (scale bar = 2 µm; *n* = 10). Data in all line or bar graphs are shown as mean ± s.e.m. For the box‐and whiskers‐graphs, minima, maxima, median, 25th and 75th percentiles are shown, with whiskers indicating the smallest and largest values. Two‐tailed Student's *t*‐tests, Welch's *t*‐tests, ANOVA, and Kruskal–Wallis tests were performed. ^*^ *p* < 0.05; ^**^ *p* < 0.01.

**Figure 8:**
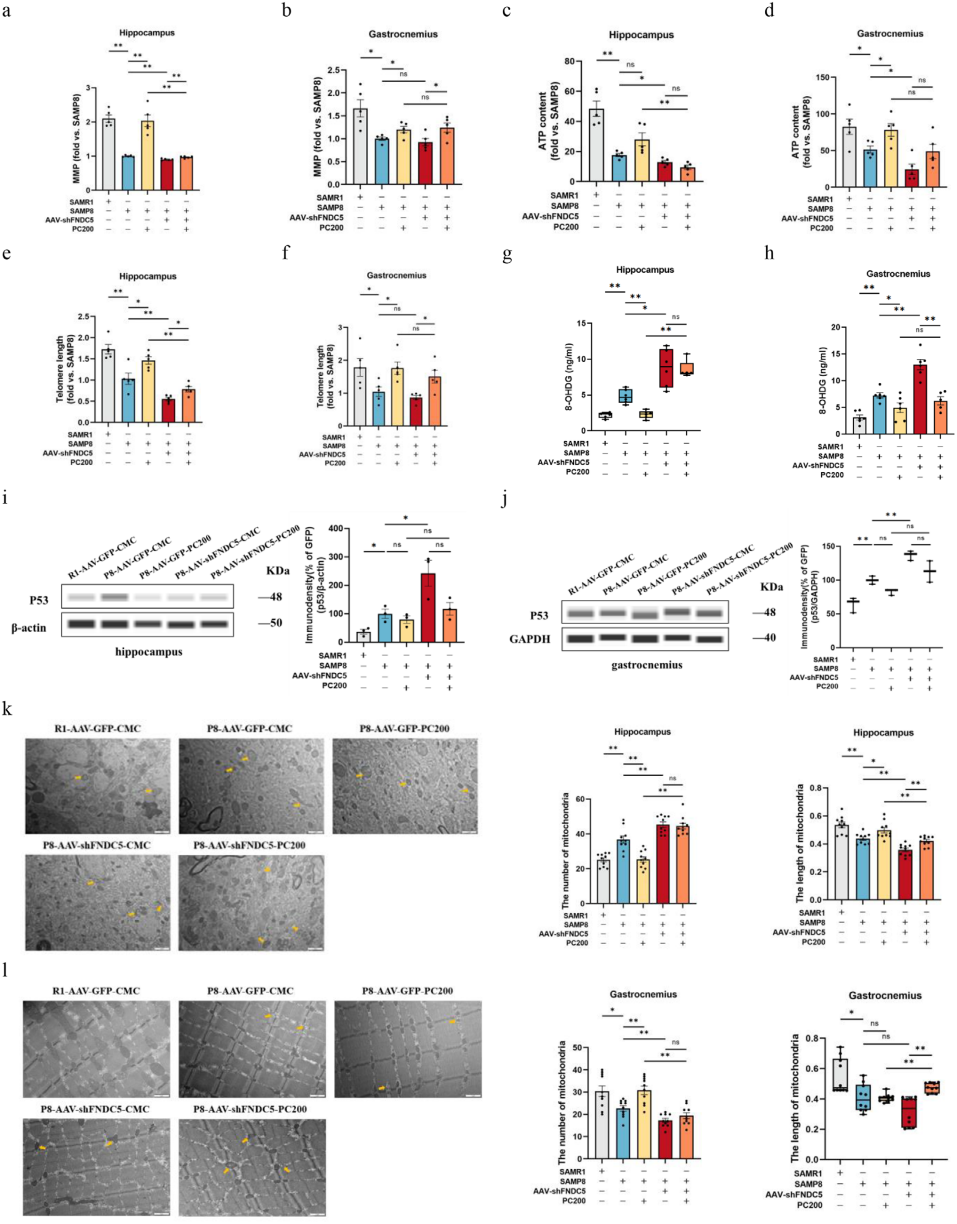
FNDC5 deficiency impaired mitochondrial function and shortened telomere length in the hippocampus and muscle. a–j) MMP (a), ATP content (c), telomere length (e), protein level of 8‐OHDG (g), and P53 (i) in the hippocampus. MMP (b), ATP content (d), telomere length (f), the protein level of 8‐OHDG (h) and P53 (j) gastrocnemius. *n*  =  5 per group. k) Represents mitochondrial number and length in the hippocampus. Abnormal mitochondria (yellow arrows) from the hippocampus (scale bar = 2 µm; *n* = 10). l, Represents mitochondrial number and length in gastrocnemius. Abnormal mitochondria (yellow arrows) from the gastrocnemius (scale bar = 2 µm; *n* = 10). Data in all line or bar graphs are shown as mean ± s.e.m. For the box‐and whiskers‐graphs, minima, maxima, median, 25th and 75th percentiles are shown, with whiskers indicating the smallest and largest values. Two‐tailed Student's *t*‐tests, Welch's *t*‐tests, ANOVA, and Kruskal–Wallis tests were performed. ^*^ *p* < 0.05; ^**^ *p* < 0.01.

PGC1α, like irisin, is considered an essential molecule for maintaining the telomere‐mitochondria axis. In order to further explore the potential biological effects of PC through regulation of the PGC1α‐FNDC5/irisin signaling pathway in SAMP8 mice, MMP, ATP content, telomere length, 8‐OHDG, and P53 protein expression in brain and muscle tissues were detected and analyzed with/without PC treatment in both the PGC1α knockdown model (**Figure** **7**) and the FNDC5/irisin knockdown model (**Figure** **8**).

Knockdown of PGC1α and FNDC5 respectively in the hippocampus led to the decrease of MMP, and ATP contents, shortened telomere length, and upregulated 8‐OHDG and P53 protein expression in brain tissue. However, the PC administration did not rescue these phenomena. On the contrary, the improvement of PC on MMP, ATP content, telomere length, as well as upregulation of PC on 8‐OHDG and P53 protein levels in muscle tissue could also be found after knocking down PGC1α or FNDC5 in the hippocampus (Figures 7a–j and 8a–j). In addition, transmission electron microscopy (TEM) was used to observe changes in mitochondrial ultrastructure in hippocampal tissue. The disorder of mitochondrial number and length, which predicted more abnormal mitochondria, could be found after knocking down PGC1α or FNDC5, while the rescue of PC was also partially blocked. (Figures 7k,l and 8k,l).

## 3 Discussion

MCI and SA are prevalent age‐related chronic degenerative diseases that mainly affect quality of life and longevity.^[^ ^10^ ^]^ Both historical and evolutionary perspectives suggest a strong link between muscle and the brain.^[^ ^31^ ^]^ Therefore, targeting and exploring the “brain‐muscle loop” association mechanism, identifying the key targets related to both diseases and exploring effective intervention ways are crucial for preventing cognitive decline and muscle attenuation in the elderly. Dietary nutrition is closely related to cognitive function and muscle health. It has been reported that nutrition could significantly influence on myocerebral function and offers an inexpensive, non‐invasive avenue for intervention.^[^ ^32^ ^]^ Therefore, dietary substances, which could regulate the key targets involved in both cognitive function and muscle health, may be the key to preventing these two diseases.

In our cohort data, through random forest models, we observed that SMM and SMI ranked in the top ten features for predicting MoCA. MoCA, in turn, was found to contribute second best to the prediction of SMM, only after energy. These results suggest a strong association between skeletal muscle mass and cognitive function, which is consistent with previous findings. Several meta‐analyses have shown that sarcopenia is a risk factor for cognitive decline.^[^ ^33^, ^34^ ^]^ Similarly, meta‐analyses have also demonstrated that elderly individuals with cognitive impairment and falls were 1.88 times more likely to develop sarcopenia than those without cognitive impairment.^[^ ^35^ ^]^ Fritz NE, et al.^[^ ^36^ ^]^ synthesized multiple studies and determined that grip strength could serve as an early indicator of future cognitive decline. Additionally, their findings showed that baseline cognitive function was found to predict grip strength at follow‐up 1–7 years later, suggesting that individuals with lower MMSE scores might experience a greater decline in grip strength over time. These studies suggest that muscle function and cognitive function might mutually promote progress, and therefore exploring the key mechanisms by which they interact might provide opportunities to simultaneously halt their progression and find effective interventions. Lecithin could be found to have a good predictive ability for MoCA, SMM, and FFMI, which was consistent with relevant studies. For instance, a prospective cohort study of 2497 middle‐aged men found that higher dietary lecithin intake was associated with a reduced risk of developing dementia.^[^ ^22^ ^]^ In targeted lipidomics analysis of erythrocytes, which is the response to long‐term dietary lipid composition, PC enriched with PUFA was significantly lower in the MCI and SA comorbidity groups compared to the control group. As an ideal dietary source of healthy PUFA, PC can provide PUFA to improve cognitive function and muscle health without the risk of excess fat consumption. Multiple studies have demonstrated that PC enriched with PUFAs plays a key role in supporting cognitive function.^[^ ^37^, ^38^, ^39^, ^40^ ^]^ Our study provides further evidence that PCs carrying unsaturated fatty acids might serve as important factors influencing the progression of cognitive impairment and sarcopenia.

Given these notable findings from our observation study, we then conducted a 24‐week intervention of unsaturated fatty acids‐rich lecithin in elderly individuals to further establish causality. In the end, compared with the placebo group, MoCA scores significantly increased in the two lecithin intervention groups, which is consistent with our previous findings of cognitive function improvements in relation to lecithin. An interventional study in 8‐month‐old SAMP8 mice with dementia showed that a 2‐month intervention with feeds rich in DHA‐PC and DHA‐PS could alleviate cognitive decline and biological damage and protect the brain.^[^ ^37^ ^]^ Injection of lecithin has also significantly alleviated the atrophy of soleus muscle in rats due to short‐term gravity sickness and altered the stiffness of soleus muscle fibers.^[^ ^28^ ^]^ In addition, a study in Japan found that high‐dose (1.2 g d^−1^) and low‐dose (0.6 g d^−1^) lecithin supplements for eight weeks in middle‐aged women (40–60 years old) can significantly improve menopausal symptoms and lower cardiovascular diastolic blood pressure and cardiopulmonary index.^[^ ^41^ ^]^ The above studies point to the potential for lecithin to improve muscle and cerebral perfusion status. We found that there was a significantly lower downward trend in CBF in the low‐dose group compared with the placebo group. However, we only observed a mediating trend in SMI and SMM in the lecithin intervention groups, which might be related to the short intervention interval.

We found that plasma irisin levels of both the high‐ and low‐dose intervention groups were significantly upregulated, suggesting that lecithin upregulates irisin expression in the human body. This is in line with a previous report of an association between triglyceride metabolites with serum irisin levels in Korean adolescents.^[^ ^29^ ^]^ There is also evidence that FNDC5/irisin can heal synaptic function and damage to pattern separation/context discrimination, suggesting that irisin may be an important small molecular active substance to improve muscle atrophy and cognitive function.^[^ ^14^, ^15^ ^]^ Further exploration is needed to understand the mechanism of PC in the above process.

The conclusions drawn from population studies are fully supported by the results of animal experiments. SAMP8 mice have been reported to exhibit progressive neurodegenerative alterations between 6 and 12 months of age,^[^ ^42^, ^43^ ^]^ and spontaneously develop a pre‐sarcopenic state at 8 months of age, resulting in overt sarcopenia at 10–12 months of age.^[^ ^44^ ^]^ In line with previous studies, we observed declines in physical performance and cognitive function in this mouse strain at 9 months of age. Similarly, Tarantini et al.^[^ ^45^ ^]^ demonstrated that C57BL/6J aged mice at the age of 24 months exhibited a notable decrease in CBF and experienced impairments in cerebral microvascular function. This provides strong evidence that cerebrovascular function decreases with age, which further contributes to impairments in learning and memory.

We also found that PC administration for 8 weeks improved the learning, memory, and muscle function in mice. Mouse micro‐ultrasound imaging and MRI results further confirmed that PC improved CBF, PWV, and blood flow velocity in mice. Consistent with findings from our population studies, the PC intervention successfully raised circulating irisin levels in mice. Multiple lines of evidence have demonstrated that^[^ ^14^, ^15^, ^46^ ^]^ individuals with impaired cognitive and muscular function exhibit decreased concentrations of FNDC5/irisin in the hippocampus and muscle. Our experiment also observed this phenomenon, and the PC intervention was found to improve this decline. Moreover, the dose of 200 mg kg^−1^ was found to be more effective than 100 mg kg^−1^ in improving both cognitive functions and sarcopenia in mice.

The analysis of the transcriptome in mice indicated that differentially expressed genes (DEGs) were continuously upregulated or down‐regulated during the PC intervention. The biological functions of DEGs involve the regulation of neurotransmitter levels, learning, memory, cognition, muscle regulation, mitochondrion organization, pro‐inflammatory cytokine, and chemokine production, and immune activation, which is consistent with previously identified risk factors of AD.^[^ ^47^, ^48^, ^49^, ^50^, ^51^ ^]^ Based on the KEGG database, the significant pathway analysis revealed that intervention with PC in SAMP8 mice altered signaling pathways linked to neurodegenerative illnesses and immune inflammation in the brain. Notably, PGC1α is involved in various biological processes and relevant pathways (REF). In addition, FNDC5 is a significant downstream gene of PGC1, and its biological functions and key pathways are currently under exploration. Relevant studies have shown that FNDC5 plays a crucial role in regulating cognitive function,^[^ ^14^, ^15^ ^]^ muscle function,^[^ ^46^ ^]^ mitochondrial function,^[^ ^52^ ^]^ and inflammation levels.^[^ ^53^ ^]^ These biological functions correspond to the aforementioned up‐regulation of PGC1α. This suggests that PGC1α‐FNDC5/irisin may have a vital role in the neuro‐ and muscle‐protective effects of PC.

In the present study, AAV9 was used to knock down FNDC5/irisin in the hippocampus of SAMP8 mice. As expected, inhibition of FNDC5/irisin in the brain led to cognitive impairment and muscle attenuation. This is consistent with other findings.^[^ ^14^, ^15^, ^46^ ^]^ It should be noted that Islam et al.^[^ ^14^ ^]^ conducted various tests such as rotarod, grip strength, treadmill gait analyses, and the open field test (OPF) on 13‐week‐old F5KO mice and found that the performance of F5KO mice was comparable to that of WT mice. However, Guo et al.^[^ ^46^ ^]^ used 5‐month‐old F5KO mice to observe muscle‐related indexes and found a reduction in muscle weight, muscle mass, grip strength, etc. In combination with our study, these results indicate that FNDC5 deficiency accelerates muscle deterioration in aged mice, but does not have the same impact on young mice.

More importantly, a novel phenomenon attracted our attention that knockdown brain irisin stimulates the upregulation of muscle and plasma irisin but fails to compensate for the decline in brain irisin. This result aligns with the findings of Lourenco, Mychael V., et al.,^[^ ^15^ ^]^ who reported that intraventricular injection of Aβ caused a nearly 50% reduction in hippocampal irisin in mice with learning and memory impairment, compared to controls. However, circulating irisin did not supplement the brain's deficiency, although irisin in peripheral blood did not change. It is inconsistent with the conclusion that irisin has the ability to cross the blood‐brain barrier to rescue memory impairment.^[^ ^15^ ^]^ This raises the question: What causes peripheral blood irisin cannot enter the brain in time in cognitively impaired mice with reduced irisin levels? It was compelling to observe that FNDC5/irisin knockdown exacerbated vascular damage in mice. Based on our extensive research into cerebrovascular damage and cognitive function,^[^ ^24^, ^25^, ^26^, ^27^ ^]^ we hypothesize that the reduction in irisin contributes to vascular injury both within and outside the brain, thereby affecting irisin entering the brain. Evidence has shown that FNDC5/irisin levels in the gastrocnemius and plasma were significantly downregulated in mice with middle cerebral artery occlusion (MCAO),^[^ ^54^ ^]^ suggesting a potential link between cerebrovascular injury and irisin concentration. Further questions need to be solved. Is there a substance that stimulates both muscle and brain irisin expression, while also enhancing vascular function to facilitate the crossing of irisin from peripheral circulation into the brain, which could be an ideal candidate for improving cognition and muscle health?

Building on this hypothesis, PC at a dose of 200 mg kg^−1^ injected into the stomach was conducted in the knockdown FNDC5/irisin mice. When FNDC5/irisin was removed, it prevented the brain from receiving the protective effects of PC. However, it still enhanced muscle function in mice. It is noteworthy that the protective effects of PC on cerebral blood vessels in mice disappeared following FNDC5/irisin knockdown. There is evidence that FNDC5/irisin is secreted by PGC1α stimulation and has a feedback regulatory effect on PGC1α.^[^ ^13^ ^]^ Our transcriptome results indicate the potential role of PGC1α in PC protection. Knockdown of PGC1α in the mouse brain also led to accelerated cognitive impairment and muscle attenuation, along with decreased FNDC5/irisin expression and protein levels in the hippocampus. The findings of these experiments provided an answer to our previous inquiry, indicating that reduced FNDC5/irisin levels in the brain contribute to cerebrovascular injury and hinder the protective effects of PC on cerebrovascular function, in turn impairing the ability of FNDC5/irisin to enter the brain. Similar to the detection of lecithin's protective effects, Andrade et al. showed^[^ ^55^ ^]^ that under conditions controlling for the level of exercise, eight weeks of resveratrol supplementation (500 mg d^−1^) given to type 1 obese patients aged 30–55 years with a BMI ≥30 kg m^−2^ resulted in a significant upregulation of FNDC5 mRNA compared to the placebo group, and this result was confirmed in animal experiments.

It has been reported that decreased PGC1α in the brain tissue of AD patients may be related to mitochondrial dysfunction.^[^ ^56^ ^]^ Furthermore, Rana et al.^[^ ^57^ ^]^ discovered that the relative length of telomeres could be predicted by age and plasma irisin levels in healthy individuals. Additional evidence suggests that telomere shortening and associated DNA damage triggers the activation of p53 and binds to PGC1α and PGC1β promoters to suppress PGCs, consequently reducing mitochondrial biogenesis and promoting compromised energy metabolism.^[^ ^58^, ^59^ ^]^ This implies that PGC1α‐FNDC5/irisin plays crucial roles in regulating the telomere‐mitochondrial axis. We studied the effects of PC intervention on various factors related to mitochondrial function and telomere length in mice. Our findings revealed that the protective effects of PC on hippocampal mitochondria and telomeres were dependent on the presence of PGC1α and FNDC5/irisin. The same protective effects were observed in muscle. Additionally, the crosstalk is still in the mechanism of PC. The obvious protective effect of PC on brain mitochondria is not only by the brain‐derived irisin but also partly by the myocyte‐derived irisin transported from circulation. When lacking of PGC1α and FNDC5/irisin within the brain, the neuro‐protective effects of PC are lost probably the reason for damaged cerebral vascular blocking the brain entrance of myocyte‐derived irisin, which the cerebrovascular injury might be aggravated by the absence of irisin. However, PC intervention still has a protective effect on muscles.

## 4 Conclusion

Our study opens a new avenue for safeguarding cognition and muscle health, averting disability in older age, and treating age‐related pathologies through lecithin supplementation, which is derived from natural sources and possesses pronounced efficacy. It serves as a promising non‐pharmacological intervention for the crosstalk of muscle and cognitive health. The protective effects of lecithin demonstrated in our investigations of both humans and mice provide strong support for further development of PC interventions, which may aid in promoting longevity.

## 5 Experimental Section

### Population Research—Participants

The current study included participants 65 years of age or older from the Effect of Dietary Nutrition on the Cognitive Function and Sarcopenia in middle‐aged and elderly People (EDNCS) cohort (ChiCTR2100054969). The EDNCS took place between 2020 and 2023 in multiple community‐based health centers and hospitals in Beijing, Jincheng of Shanxi Province, and Linyi of Shandong Province in China. All participants in this study were listed in the community census or village registry and had resided in the research center for at least one year preceding the survey date. Inclusion criteria included: >65 years of age at the time of recruitment, able to complete a full interview, and voluntary agreement to participate in the study. The exclusion criteria included: cognitive impairment caused by depression, stroke, traumatic brain injury, or other severe organ dysfunction; refusal to participate in the study; current use of medication or dietary supplement to improve cognitive function; and uncorrected visual or hearing impairment. A total of 1970 participants were included and underwent face‐to‐face interviews at baseline, consisting of the collection of sociodemographic information, medical history of chronic diseases, cognitive assessment, dietary surveys, and measurement of body composition. Subsequently, two follow‐up visits were subsequently conducted. Informed consent was obtained from all enrolled participants.

Participants aged 65 years or older come were enrolled from EDNCS (ChiCTR2100054969) between August 2020, and December 2021 to participate in an RCT study, examining the effects of phosphatidylcholine among participants 65 years of age or older. Inclusion criteria and exclusion criteria were the same as those used in the queue study. At both baseline and 24 weeks, participants underwent cognitive function assessment, body composition measurement, dietary assessment, and cerebral blood flow measurement. A total of 292 participants completed the intervention and were assigned to one of three groups: low‐dose phosphatidylcholine (N = 100), high‐dose phosphatidylcholine (N = 95), or placebo (N = 97). The study was conducted in accordance with the principles of the Declaration of Helsinki and ethically approved by the Ethics Committee of Capital Medical University (Z2019SY052, Z2023SY023). The population study profile is available in Figure S11 (Supporting Information).

### Population Research—Randomized Control Trial (RCT)

After baseline data collection (demographic, anthropometric, outcome measures) and blood sample collection, participants were randomly assigned to a low‐dose phosphatidylcholine group (N = 100), a high‐dose phosphatidylcholine group (N = 100), or placebo group (N = 100). The participants, study teams, and outcome assessors were blinded to treatment assignment. All participants took 2.4 or 4.8 g lecithin (or look‐alike placebo) daily for 24 weeks. The high‐dose and low‐dose soy lecithin and placebo tablets, indistinguishable in shape, weight, and color, were provided by Swisse Wellness Pty Ltd (Australia). The tablet contained phospholipids in the form of phosphatidylcholine, 8.7%; phosphatidylethanolamine, 11%; and phosphatidylinositol, 21%. Participants were instructed to take two or four tablets per day. Medication adherence was assessed by collecting the supplement packages from the participants. Each participant was assigned a sequential number and received supplement packages with corresponding numbers. The actual contents of each package, whether it contained high‐dose or low‐dose soy lecithin or a placebo, were known only to the manufacturers. As a result, both the participants and the investigators were unaware of the allocation. Safety was evaluated based on the occurrence of treatment‐emergent adverse events reported by the participants. Outcome assessments were conducted at the medical center by a trained study team blinded to whether lecithin or placebo was administered. A schematic of the RCT study is available in Figure S12 (Supporting Information).

### Population Research—Cognitive Function Assessment

Neuropsychological tests were done to determine the cognitive status of the participants at baseline and at each follow‐up visit. Global cognitive function was assessed using the Chinese version of the Mini‐Mental State Examination (MMSE) and Montreal Cognitive Assessment (MoCA). Mild cognitive impairment was diagnosed on the consensus of at least two neurologists, according to the Peterson criteria.^[^ ^60^ ^]^

### Population Research—Body Composition and Sarcopenia Assessment

Skeletal muscle mass was estimated by bioelectrical impedance analysis (BIA) (InBody 720 analyzer, InBody Co., Ltd., South Korea). Electrical current was supplied by electrodes on the tips of both toes and fingers. Subjects stepped barefoot on the weighing platform with their heels on the rear electrodes and the front part of their feet touching the front electrodes. They stood still without bending their knees. The subjects also grasped the grips with both hands. When the measurements were completed, the analyzer displayed the resistance. Skeletal muscle mass (SMM, kg), appendicular skeletal muscle mass (ASM, kg), and fat‐free mass (FFM, kg) were all measured. The skeletal muscle mass index (SMI) was obtained by dividing ASM by height squared (m^2^), and the fat‐free mass index (FFMI) was obtained by dividing FFM by height squared (m^2^).

Sarcopenia was assessed based on the Asian Working Group for Sarcopenia (AWGS) 2019 criteria.^[^ ^11^ ^]^ Sarcopenia was diagnosed when low skeletal muscle index (SMI) plus low grip strength or low physical function were detected. Hand grip strength (kg) was measured in the dominant hand and non‐dominant hand when the participant squeezed a handgrip dynamometer (Yagami Co, Tokyo, Japan) as hard as possible. The maximum value of 3 consecutive measurements performed in this study was recorded. The cut‐off points for low grip strength for men and women were <28 and <18 kg, respectively. SMI was calculated using the appendicular skeletal muscle mass of arms and legs. The cut‐off points of SMI for sarcopenia were <7.0kg m^−1^ 2 for males and <5.7kg m^−1^ 2 for females. A 6 m timed distance was used to evaluate the usual gait speed of natural walking. The 5‐chair stand test (5‐CST) asks participants to stand up from a chair and sit down five times as quickly as possible. The lower level of physical function was defined as gait speed<1.0m s^−1^ or 5‐CST≤12s for both males and females.

### Population Research—Dietary Assessment

Dietary information was collected by the Food Frequency Questionnaire (FFQ) of 2002, according to the China National Nutrition and Health Survey (CNHS 2002).^[^ ^61^ ^]^ Energy and nutrient intakes were calculated according to the China Food Composition Database (Version 6).^[^ ^62^ ^]^

### Population Research—Blood Sample Collection and Targeted Lipidomic for Cohort Sample

Fasting blood samples were collected in the morning from participants who had fasted from 8 p.m. the night before (fasting time ≥ 10 h). **Metabolite Extraction**: All blood cell samples were vortexed for 60 s with 400 µL water, and then the samples were homogenized at 45 Hz for 4 min, and sonicated for 5 min in an ice‐water bath. The homogenization and sonication circle was repeated three times. 50 µL of homogenate was mixed with 150 µL water, and then 480 µL of extraction solution containing internal standard was added. After 60 s of vortexing, the samples were sonicated for 10 min in the ice‐water bath. Then the samples were centrifuged at 3000 rpm for 15 min at 4 °C. 250 µL of supernatant was transferred to a fresh tube. The rest of the sample was added with 250 µL of MTBE, followed by vortex, sonication, and centrifugation, after which another 250 µL of supernatant was taken out. This step was repeated once. The supernatants were combined and dried in a vacuum concentrator at 37 °C. The dried samples were then reconstituted in 100 µL of resuspension buffer (DCM: MeOH: H_2_O = 60: 30: 4.5), vortexed for 30 s, and sonicated for 10 min in the ice‐water bath. The constitution was then centrifuged at 12 000 rpm for 15 min at 4 °C and 40 µL of supernatant was transferred to a fresh glass vial for LC/MS analysis. The quality control (QC) sample was prepared by mixing an equal aliquot of the supernatants from all of the samples. **LC‐MS/MS Analysis**: the UHPLC separation was carried out using a SCIEX ExionLC series UHPLC System. The mobile phase A consisted of 40% water, 60% acetonitrile, and 10 mmol L^−1^ ammonium formate. The mobile phase B consisted of 10% acetonitrile and 90% isopropanol, and 10 mmol L^−1^ ammonium formate. The column temperature was 40 °C. The auto‐sampler temperature was 6 °C and the injection volume was 2 µL. AB Sciex QTrap 6500+ mass spectrometer was applied for assay development. Typical ion source parameters were: IonSpray Voltage: +5500/‐4500 V, Curtain Gas: 40 psi, Temperature: 350 °C, Ion Source Gas 1:50 psi, Ion Source Gas 2: 50 psi, DP: ±80V. **Data preprocessing and annotation**: Biobud‐v2.07 Software was employed for the quantification of the target compounds. The absolute content of individual lipids corresponding to the IS was calculated on the basis of peak area and the actual concentration of the identical lipid class internal standard (IS).

### Population Research—Targeted Lipidomic for RCT Sample

**Metabolite Extraction** and **data preprocessing and annotation** are the same as above. A SCIEX Triple Quad 7500 mass spectrometer was applied for assay development in **LC‐MS/MS Analysis**. Typical ion source parameters were: IonSpray Voltage: +3500/−3000 V, Curtain Gas: 50 psi, Temperature: 400 °C, Ion Source Gas 1:50 psi, Ion Source Gas 2: 50 psi. Before mass spectrometry analysis, the optimal Q1 / Q3 pair was selected for each target lipid, and its MRM parameters were optimized. Ion pairs were used for qualitative and quantitative analysis. The specific data are shown in the results table.

### Population Research—Enzyme‐Linked Immunosorbent Assay (ELISA) of Human Plasma

Irisin blood concentrations were measured in duplicate using the ELISA method (EK‐067‐29, Phoenix Pharmaceuticals, Inc.).

### Population Research—Cerebral Blood Flow Measurement

A 16.0 MHz linear array transducer from a computed sonography system (ACUSON P500, Siemens Medical Solutions USA, Inc.) was used to take flow volume measurements in the extracranial arteries. The site where blood flow was easiest to assess was used for measurements. In the extracranial arteries, the examination site was therefore 1.0–1.5 cm away from the bifurcation in ICA and ECA, ≈1.5 cm below the carotid bulb in the CCA, and usually at the C4–C5 intertransverse area of the vertebral artery. The luminal diameter (d) of each artery was measured on a magnified B‐mode image between the two endothelial layers and perpendicular to the vessel's course. At the same site in each vessel, a pulsed Doppler spectrum was recorded with a sample volume covering the entire luminal width. The Doppler frequencies were angle‐corrected by adjusting the angle between the Doppler beam and the course of the vessel in 1‐degree steps. Angle‐corrected, time‐averaged mean velocity (TAV) was measured over at least three complete cardiac cycles. The angle‐corrected TAV was the time‐integrated mean of the mean frequencies and hence was equivalent to the average velocity of all cellular elements passing the sample volume. It was directly proportional to the intravascular flow volume transport. The flow volume (FV) in each artery was determined by calculating the product of the angle‐corrected TAV and the cross‐sectional area (A) of the circular vessel with the formula:

(1) $$\mathrm{FV}=\mathrm{TA}V^{*}A=\mathrm{TA}V^{*}\left[\left(d/2\right)2^{*}\Pi \right]$$

Global CBF volume was defined as the sum of flow volumes in the ICA and VA of both sides.

### In Vivo Study—Animal

Three‐month‐old male SAMP8 and SAMR1 mice (18–20 g) were purchased from the laboratory animal center of the Peking University Health Science Center (Beijing, China). Six‐month‐old male APPswe/PSEN1dE9 (APP/PS1) double transgene mice and C57BL/6J mice (18–20 g) were obtained from Beijing HFK Bioscience Co., Ltd. (Beijing, China). The mice were housed individually (one mouse per cage) in the animal center of the Capital Medical University, with a temperature of 22 ± 2 °C and humidity of 50% ± 10%. Mice had free access to food and water under a controlled 12 h light‐dark cycle. All the animals used in the experiments were six months old at the start. All experimental procedures were conducted in accordance with the National Institutes of Health Guide for the Care and Use of Laboratory Animals, 8th edition, and were approved by the ethics committee of Capital Medical University (AEEI‐2019‐138). A schematic of the experimental layout is available in Figure S12 (Supporting Information).

### In Vivo Study—Stereotactic Injection of Adeno‐Associated Virus (AAV)

Six‐month‐old mice were anesthetized with isoflurane and placed on a stereotaxic apparatus and then were sterilized with iodophor, and the scalp was incised along the midline of the head. With reference to the Allen Reference Atlas, the Franklin and Paxinos Mouse Atlas, Adeno‐associated virus particles (1.12−1.40 × 10^13^) expressing shRNA against murine FNDC5, PGC1α or Luciferase (control) were stereotaxically injected into the right lateral ventricle of SAMP8 or SAMR1 mice (coordinates relative to Bregma: 0.3 mm AP; 1.0 ML; 2.2 mm DV). Adeno‐associated virus particles (4.92−5.50 × 10^12^) harboring FNDC5 (AAV‐FNDC5) constructs were i.c.v.‐injected as described above. 3 µL was injected of a virus into the right lateral ventricle at 0.05 µL min^−1^. The syringe was not removed until 10 min after the end of infusion to allow diffusion of the virus. The skin was sutured, and mice were placed beside a heater for recovery. After injection, mice were allowed 3–4 weeks of recovery. AAV were provided by Obio Technology (Shanghai, China).

### In Vivo Study—Intragastric Administration

PC was obtained from Sigma‐Aldrich (cat #429 415; St. Louis, MO). Seven‐month‐old mice received PC (dissolved in 0.5% CMC‐Na, 100 or 200 mg kg^−1^ at the same time every day) or vehicle (0.5% CMC‐Na) daily by oral gavage for 8 weeks.

### Behavioral Testing—Novel Object Recognition (NOR)

The experimental apparatus consisted of an open plastic box (50 cm × 50 cm × 40 cm). The test included two trials. After habituation to the test area for 5 min, two identical objects were placed at opposite corners of the area. The mouse was allowed to explore for 10 min for the first trial. 60 min after the first trial, one of the objects (a familiar object) was exchanged for a novel object. The novel object was different in shape and color but was consistent in height. In the second trial, the mouse was returned to the test area with the familiar and novel object for 10 min. The discrimination index (DI) was calculated as the time spent exploring the novel object / the time spent exploring the familiar object + the time spent exploring the novel object.

### Behavioral Testing—Morris Water Maze (MWM)

To assess the hippocampus‐dependent learning and memory abilities of the mice, the MWM test was performed. The MWM apparatus consisted of a blue circular pool with a diameter of 120 cm filled 2/3 with water (25 °C ± 1) containing nontoxic ink and a circular platform (14 cm in diameter) submerged 1.5 cm below the water surface. Each mouse was trained to find the hidden platform within 60 s for 5 consecutive days with 3 trials per day. A 60 s probe trial was performed at the indicated time points after the training. The Probe Test was the second phase of the experiment. During this phase, the platform was removed and the mice were started from the quadrant opposite of the platform quadrant. The time each mouse needed to find the platform within 60 s was defined as the escape latency. If a mouse did not find the platform successfully, the latency was recorded as 60 s, and the mouse was gently guided to the platform and allowed to stay on it for 10 s. The latency was recorded.

### Behavioral Testing—Rotarod Test

The rotarod apparatus (Beijing Zhongshidichuang Science and Technology Development Co., Ltd, China) consisted of a rod (30 mm in diameter and 62 mm wide) flanked by two large round plates (40 cm in diameter). The speed of rotation was increased from 4 to 40 rpm over 5 mins and then remained at 40 rpm for an additional 300 s, and maintained for 300 s. The time taken for each mouse to either fall off the rod or cling onto it for one complete rotation was recorded. Mice were tested for two more consecutive days, two trials per day. Maximum running time was used for further analysis.

### Behavioral Testing—Grip Test

To measure the muscle strength of mice, the commercially available Grip Strength Meter (Beijing Zhongshidichuang Science and Technology Development Co., Ltd.) was used. For each trial, the mouse was held by the tail and placed, gently, so that its forepaws or four paws grabbed the bar. Then, the mouse was smoothly pulled back until it released the bar, and the forepaws or four‐paws grip in Newtons (N) was recorded. Five trials were carried out and averaged.

### Behavioral Testing—Hanging Grid Test

In this assay, inverted hanging time was measured. A 45 × 45 cm grid (bar thickness, 1.5 mm; mesh, 18 mm) was placed on a 55 cm‐high frame, and a 5 cm‐thick cushion was placed under the grid. The distance between the grid and the cushion was 50 cm. Each mouse was placed at the center of the grid and then turned the grid upside down with the mouse head declining first. Hanging time was recorded as the time until the mice fell. Each mouse was tested thrice with a >30 min interval between tests, and the hanging time was recorded and averaged.

### Body Composition Measurements of Mice

Lean mass was determined using magnetic resonance imaging, ECHO‐MRI (Echo Medical Systems, TX, USA). Mice were individually placed in the MRI machine and three measurements were performed on each mouse. The mean values of lean mass were computed.

### Magnetic Resonance Imaging (MRI)

Imaging was performed on a 7.0 Tesla small animal MRI system with 450 mT m^−1^ gradient amplitude and a 4500 T m^−1^ s^−1^ slew rate (Biospec 70/30, Bruker). The animals were anesthetized with isoflurane in oxygen and immobilized in the MRI using a nose cone and bite ring. All mice were initially anesthetized with 5% isoflurane in oxygen and then transferred to an animal holder within the magnet where they were maintained on 1.5–2% isoflurane in oxygen during imaging. Respiration rate was monitored using a respiration pad (Small Animal Instruments) and maintained at ≈30–35 breaths per minute to minimize motion artifacts during imaging. Regional CBF was monitored by arterial spin labeling (ASL). Region of interest (ROIs) was first placed in the bilateral hippocampus and whole brain on the T1 image and then copied to the CBF map to acquire corresponding CBF.

### Vascular Doppler Ultrasound Examination

Doppler ultrasonography was performed of bilateral carotid arteries to assess vascular function and structure on the mice using the Vevo 2100 Imaging System (FUJIFILM VisualSonics Inc., USA) before they were sacrificed. All indicators were monitored from at least 3 consecutive cardiac cycles, including PWV, and blood flow velocity, and these indicators were averaged over consecutive cardiac cycles.

### Tissue Preparation

Brain samples were removed and separated along the middle sagittal sulcus with the animals deeply anesthetized. Hippocampal regions were dissected from each brain and immediately stored at −80° C for biochemical assays. Three mice per group were perfused with cold normal saline via the ascending aorta, the whole brain was placed in 4% paraformaldehyde, dehydrated in 15% and 30% sucrose solutions, and then frozen for 10‐µm‐thick sections for SA‐β‐gal staining. The gastrocnemius muscles were removed from both hind limbs. After weighing the muscle mass, one gastrocnemius muscle (from the right hind limb) was immediately frozen in liquid nitrogen and stored at −80 °C for biochemical assays.

### Mitochondrial Ultrastructure

Freshly harvested hippocampus and gastrocnemius tissue from mice was processed for transmission electron microscopy. Briefly, blocks of tissues (1mm^3^) were fixed with 2.5% glutaraldehyde in 0.1 m cacodylate buffer at 4 °C for 4 h and post‐fixed with 1% osmium tetroxide at room temperature for 1 h, followed by dehydration, embedding in epoxy resin, and polymerization at 60 °C overnight. Ultra‐thin sections (100 nm) were stained with uranyl acetate and Reynold's lead citrate and photographed with a transmission electron microscope (JEM‐1400plus, Hitachi, Tokyo, Japan). Ten randomly chosen images per group were quantified for mitochondrial number and diameter using Image J.

### Quantitative Real‐Time PCR (qRT‐PCR)

The total RNA of tissues was extracted with TRIzol reagent (Invitrogen). The quality of RNA was assessed with a 260/280 nm absorption ratio; RNA was used only when the ratio was 1.8–2.0. In brief, total RNA (1 µg) was reverse‐transcribed into cDNA using Reverse Transcription Reagent kits (ThermoFisher, USA), according to the manufacturer's instructions. The PCR reactions were run on a CFX Connect Real‐Time PCR Detection System (Bio‐Rad, Hercules, CA, USA). Primers were designed specifically based on the NCBI database, the forward and reverse primer sequences used in this study are shown in **Table** **1**. The reactions were conducted in a total volume of 20 µL comprising 10 µL of Master Mix with SYBR Green, 0.4 µL of each primer (10 µm), 1 µL of sample cDNA, and 8.2 µL deionized water. The reactions were under the following conditions: 95 °C for 10 min, 40 cycles of 95 °C for 10 s, 60 °C for 20 s and 72 °C for 10 s. β‐actin and GAPDH served as an internal reference for normalization. The data were quantified and expressed as fold‐change compared to the control by using the ΔΔCT method.

**Table 1:** The primer sequences used for qRT‐PCR.

Gene	Forward Sequence (5′‐3′)	Forward Sequence (5′‐3′)
FNDC5	GGATATACTTTACGCAGGTCGA	CGTCTGAGTTGGTATCTAGGTC
PGC1α	GGCTTTGCCATCTCTCAGCA	GAGATGGCCTGCACATGGAC

### Western Blotting and Capillary Immunoassay—Western Blotting

Proteins were extracted from hippocampus and gastrocnemius muscles, separated on 10–12% sodium dodecyl sulfate‐polyacrylamide gels, and transferred onto PVDF membranes (Millipore IPVH00010, USA), which were soaked in Tris‐buffered saline‐Tween (TBST) solution containing 5% non‐fat milk (room temperature, 1 h) to block non‐specific binding; subsequently, the membranes were exposed to primary antibodies: FNDC5 1:1000 (ab174833, Abcam), PGC1α 1:1000 (ab191838, Abcam), β‐actin 1:3000 (#4970, CST), GAPDH 1:3000 (#5174, CST). After staining overnight at 4 °C, the membranes were washed thrice with TBST and incubated with anti‐rabbit (#7074, CST) secondary antibodies at room temperature for 60 min. After washing thrice more with TBST, an enhanced chemiluminescence (ECL) reagent (AQ529, Bejing Aoqing Biotechnology Co., Ltd, China) was added, and images were obtained using Fluorchem FC 2 software and quantified using ImageJ.

### Western Blotting and Capillary Immunoassay—Capillary Immunoassay

Equal amounts of protein from the crude homogenates were analyzed using the automated simple western system, Wes (ProteinSimple, Bio‐Techne, San Jose, CA), which utilizes a capillary‐based immunoassay. The protocol described in the manual was followed to detect HTT, GFAP, and DARPP32 using 0.6 µg of sample. Quantitative analysis of protein levels were done automatically using the Compass for Simple Western Software (ProteinSimple) on electropherogram data. The peak area (using the automatic “dropped line” option in software) of each protein of interest was normalized to the peak area of the vinculin loading control. Figures showed protein bands similar to traditional western blots using the “lane view” option in the Compass software to create a blot‐like image from the electropherogram data.

### Enzyme‐Linked Immunosorbent Assay (ELISA) of Plasma, Brain, and Muscle Tissues

Brain tissues and muscle tissues were accurately weighed and then added to ice‐cold PBS at a ratio of 1: 9 by weight. The tissues were then homogenized in an ice bath, centrifuged, and the supernatant was obtained. Blood was collected in EDTA‐coated plastic tubes, and centrifuged, and plasma fractions were freshly used for ELISA. The following ELISA kits were used: Irisin ELISA kits (EK‐067‐29, Phoenix Pharmaceuticals, inc.), 8‐hydroxydeoxyguanosine (8‐OHDG) (H165‐1‐2, Nanjing Jiancheng Bioengineering Institute, China).

### SA‐β‐Gal Staining

The activity of SA‐β‐gal was investigated in accordance with the guidelines of the manufacturer utilizing a kit (C0602, Beyotime Biotechnology, China). Images were gathered randomly with a Panoramic Scan (3DHISTECH Ltd., Budapest, Hungary). The area of SA‐β‐gal‐positive cells was measured with ImageJ.

### Targeted Lipidomic

Metabolites were extracted and analyzed using the same technique detailed above for human participants.

### Quantitative Inflammation Array

Fresh brain tissues and muscle tissues were taken from the whole brain and gastrocnemius muscle, as described above. The concentration of 40 cytokines in the brain and muscle was determined using the Quantibody Mouse Inflammation Array Kit according to the manufacturer's instructions (RayBiotech, Inc., Norcross, GA, USA).

### RNA‐Sequencing

Sequencing libraries for RNA‐sequencing were prepared using the Hieff NGS Ultima Dual‐mode RNA Library Prep Kit for Illumina. Paired‐end 100 bp read sequencing was performed on a DNBSEQ‐T7 system using Combinatorial Probe‐Anchor Synthesis (cPAS). Reads of each sample were aligned to the mouse genome (NCBI build 38/mm10) using Bowtie v2.2.3. HTSeq v0.6.1 was used to count the read numbers mapped to each gene and then the TPM of each gene was calculated. DEG analysis was performed using EdgeR 3.28.1. Genes with a |log2FC| > 0.25 and P < 0.05 were included and defined as DEGs. These DEGs were further used for GO and KEGG analyses. Heatmap was produced using pheatmap (1.0.12).

### Measurement of Mitochondrial Membrane Potential (MMP), ATP, Telomere Length

MMP was evaluated by JC‐1 staining (C2003S; Beyotime, China); ATP levels were measured by the ATP Assay Kit (S0027; Beyotime, China); qRT‐PCR was used to determine changes in average telomere length of mice based on Relative Mouse Telomere Length Quantification qPCR Assay Kit (#8908, ScienCell Research Laboratories, inc., USA).

### Statistical Analysis

Statistical analyses and graphical presentations were performed using SPSS (version 21.0), R software (version 4.0.4), and GraphPad Prism (version 9.5.0).

Random forest models were configured to find the most significant variables for the prediction of MoCA, SMM, SMI, and FFMI separately. Specifically, in the prediction of MoCA, 29 variables related to skeletal muscles and blood were included in the random forest model; when predicting SMM, SMI, and FFMI, only 22 blood‐related variables were incorporated into the random forest model. A random forest model was first built based on the baseline data. For model tuning, the optimal number of trees in the random forest was determined by plotting error curves with different numbers of trees from the RF models, and the optimal mtry value which controlled the number of variables randomly sampled as candidates at each split was determined via grid search. Because all predictions were continuous variables, the increase in MSE (incMSE) was calculated to evaluate the importance of each variable. The incMSE represents the increase in MSE when a variable changes. If a variable was important, the incMSE would be large when it changes, and vice versa. As mentioned above, random forest models were built based on baseline data, completed feature importance evaluation, and used the trained RF models on the baseline data to predict MOCA, SMM, SMI, and FFMI at the second and third follow‐up visits. The importance was evaluated of lecithin for predicting MOCA, SMM, SMI, and FFMI through partial correlation plots between lecithin and MOCA, SMM, SMI, and FFMI. The above process was carried out in R (version 4.0.4).

For the intervention, the differences (Δ) were first calculated in MOCA, skeletal muscle indices, irisin, and CBF before and after intervention by subtracting the pre‐intervention values from the post‐intervention values. The differences in the pre‐and post‐intervention differences of MOCA, skeletal muscle indices, irisin, and CBF among the low‐dose group, high‐dose group, and placebo group were compared using analysis of variance (ANOVA). The Bonferroni method was used for post‐hoc analysis. Furthermore, the intervention population was divided into disease groups (MCI, sarcopenia, or MCI with sarcopenia) and healthy control groups. Multivariable regression analysis was employed to explore the interactions between different intervention methods and intervention populations. All statistical tests were considered significant at *p* < 0.05. The above process was carried out in R (version 4.0.4).

For the animal research, results were presented as the mean ± standard error of the mean and analyzed by Student's *t*‐test and one‐way analysis of variance (ANOVA) followed by least significant difference (LSD) post hoc tests. Two‐tailed Student's *t*‐tests were used to compare two groups with a Welch's correction if variances were significantly different between groups. For non‐normally distributed parameters, such as the Longest running time of the rotarod test, Mann–Whitney U tests, or Kruskal–Wallis tests were used, and the data were presented using median and interquartile range. For latency to the platform of MWM, a generalized estimating equation (GEE) was used. The above process was carried out in SPSS (version 21.0). All statistical tests were considered significant at *p* < 0.05.

## Conflict of Interest

The authors declare no conflict of interest.

## Author Contributions

Y.D.X. and W.L.X. are equal corresponding authors. X.Y.W, D.J.L., X.Y.L., and W.Z.L. contributed equally to this work and shared the first authorship. Y.D.X. and W.L.X. conceived and designed the work; X.Y.W and D.J.L. performed most of the experiments with assistance from X.W., C.Y.Q, H.N.D., and Y.F.C.; Data analyses assistance and advice was provided by R.X., H.H.M. Y.A., W.Z.L., X.W., T.T.L., M.M.D., and K.Y.; W.Z.L., X.W., T.T.L., X.Y.L., C.Y.Q., H.N.D., and J.S. collected and analyzed population data. X.Y.W., D.J.L., and Y.F.C performed behavioral studies; Y.A. and J.S. performed the MRI and Doppler ultrasound examination; X.Y.W. and D.J.L. wrote the paper; Y.D.X., R.X., and W.L.X. revised the paper.

## Supporting information



Supporting Information

## Data Availability

Research data are not shared.
