# Supplementary material for: Lecithin Alleviates Memory Deficits and Muscle Attenuation in Chinese Older Adults and SAMP8 Mice
Source: Adv Sci (Weinh). 2025 May 9;12(30):2405222. doi: 10.1002/advs.202405222 (PMC12376584; doi:10.1002/advs.202405222)
Supplement: Supplementary file 1 — Supporting Information [file ADVS-12-2405222-s001.docx]

**Supplementary figures**

| a | b |
| --- | --- |
| 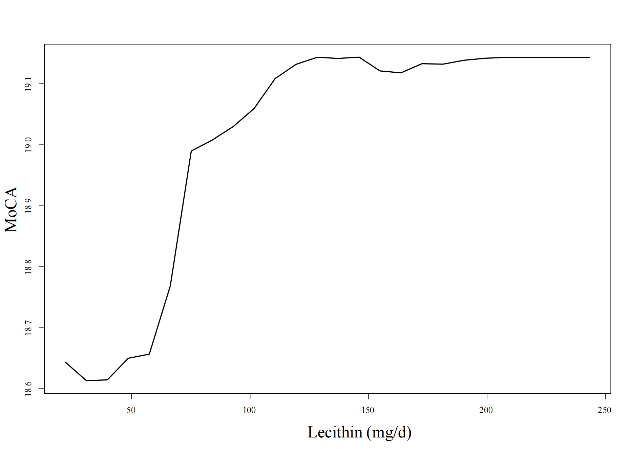 | 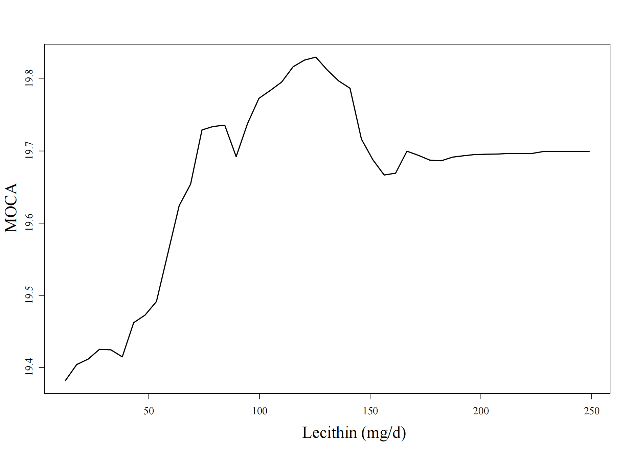 |
| c | d |
| 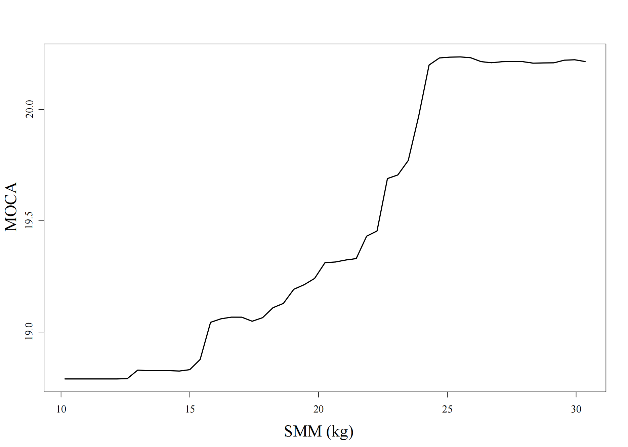 | 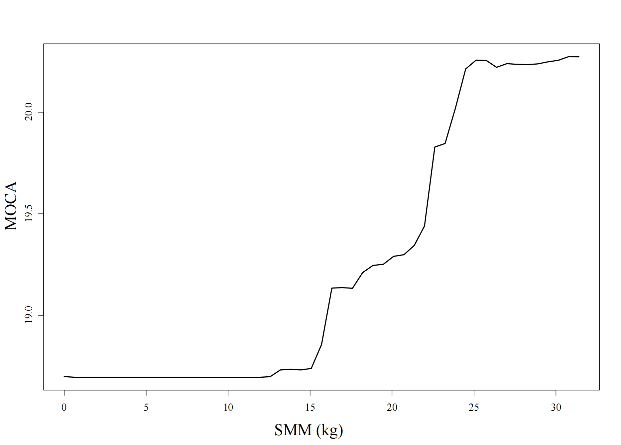 |
| e | f |
| 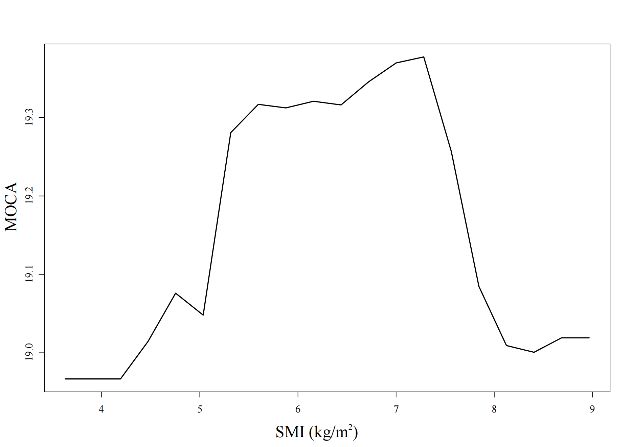 | 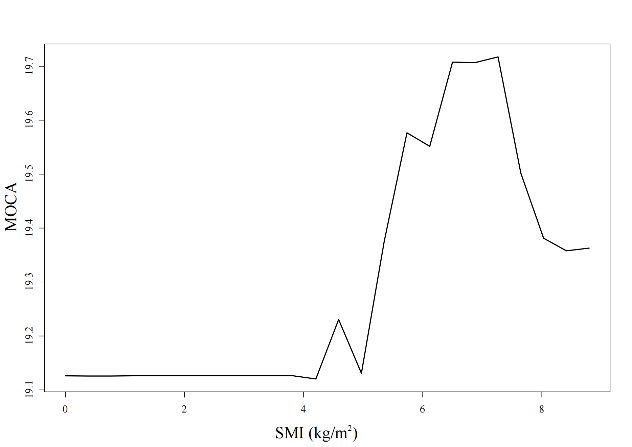 |
| **Figure S1.** Partial dependence plot (PDP) of the random forest model in the prediction of MoCA. PDP demonstrates the marginal effect of lecithin in the prediction of MoCA in the (a) second follow-up visit and (b) third follow-up visit data. PDP demonstrates the marginal effect of SMM in the prediction of MoCA in the (c) second follow-up visit and (d) third follow-up visit data. PDP demonstrates the marginal effect of SMI in the prediction of MoCA in the (e) second follow-up visit and (f) third follow-up visit data. | |

| a | b |
| --- | --- |
| 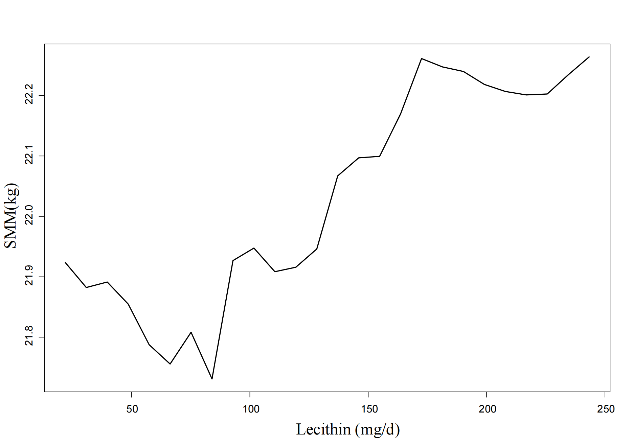 | 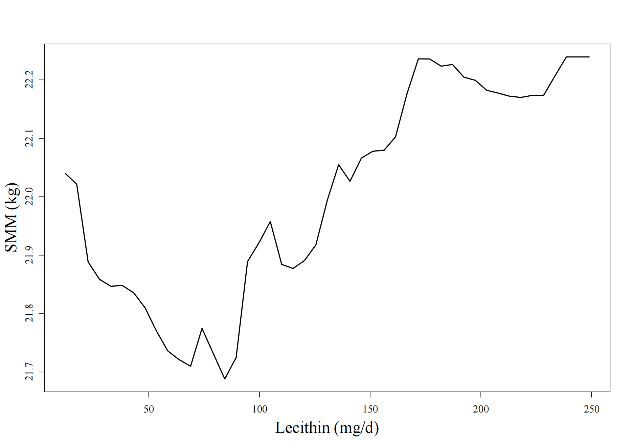 |
| **c** | d |
| 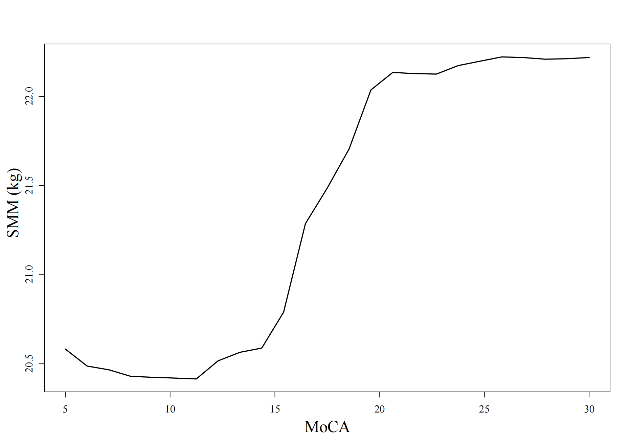 | 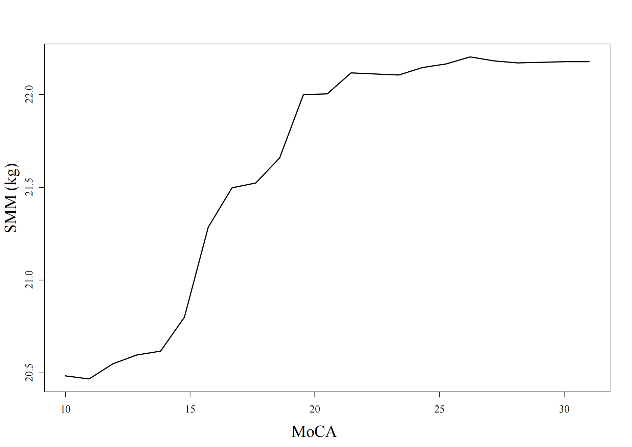 |
| **Figure S2.** Partial dependence plot (PDP) of the random forest model in the prediction of SMM. PDP demonstrates the marginal effect of lecithin in the prediction of SMM in the (a) second follow-up visit and (b) third follow-up visit data. PDP demonstrates the marginal effect of MoCA in the prediction of SMM in the (c) second follow-up visit and (d) third follow-up visit data. | |

| a | b |
| --- | --- |
| 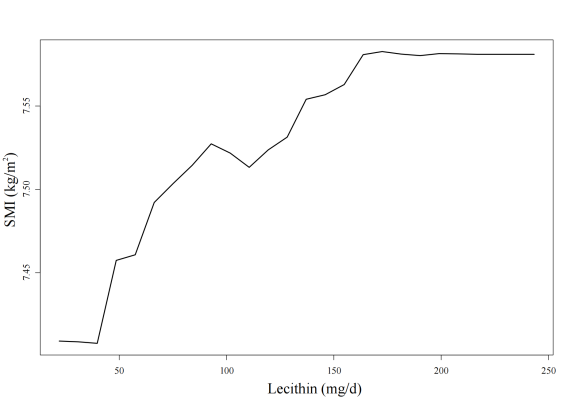 | 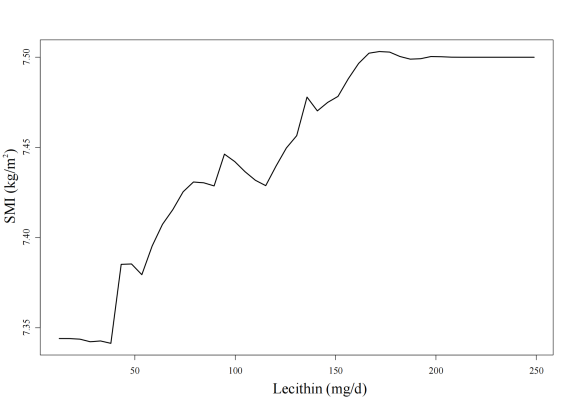 |
| **c** | **d** |
| 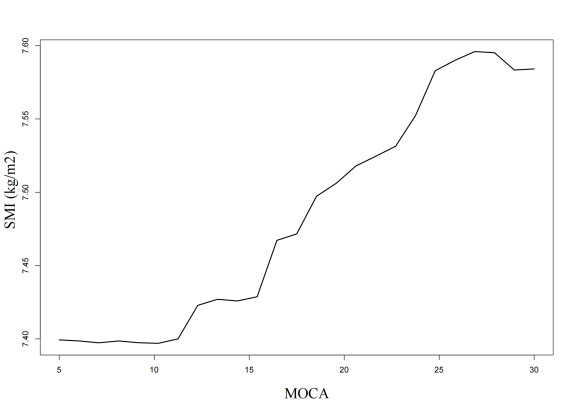 | 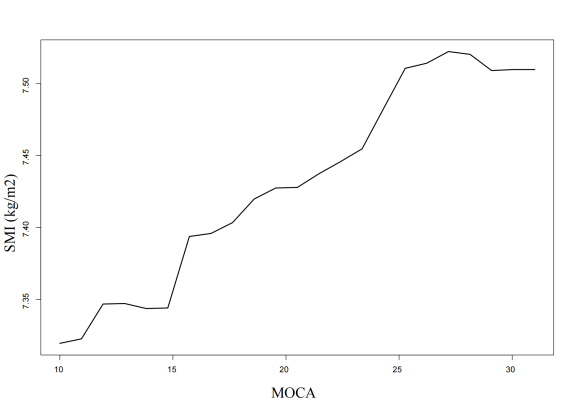 |
| **Figure S3.** Partial dependence plot (PDP) of the random forest model in the prediction of SMI. PDP demonstrates the marginal effect of lecithin in the prediction of SMI in the (a) second follow-up visit and (b) third follow-up visit data. PDP demonstrates the marginal effect of MoCA in the prediction of SMI in the (c) second follow-up visit and (d) third follow-up visit data. | |

| a | b |
| --- | --- |
| 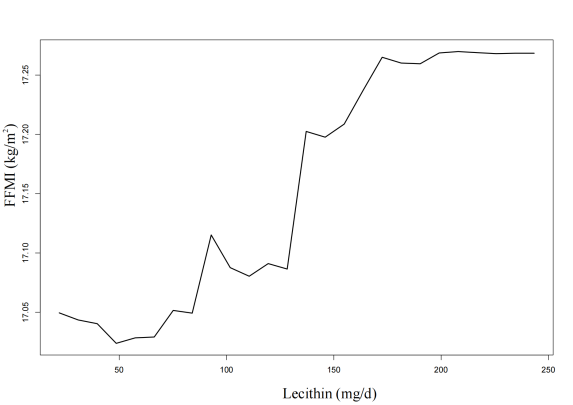 | 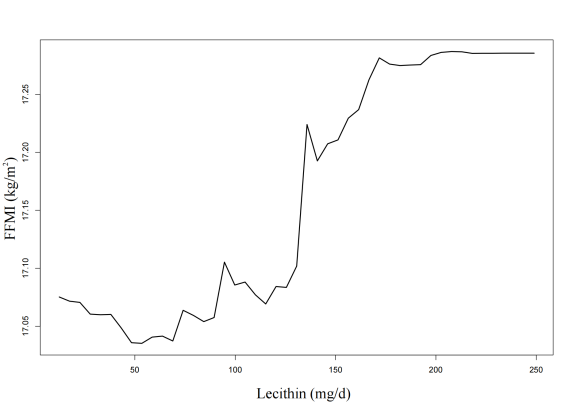 |
| **c** | **d** |
| 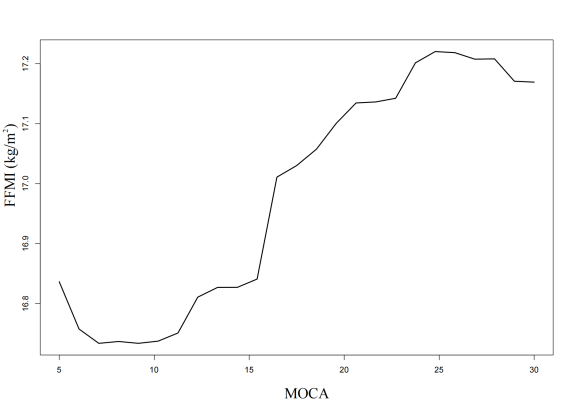 | 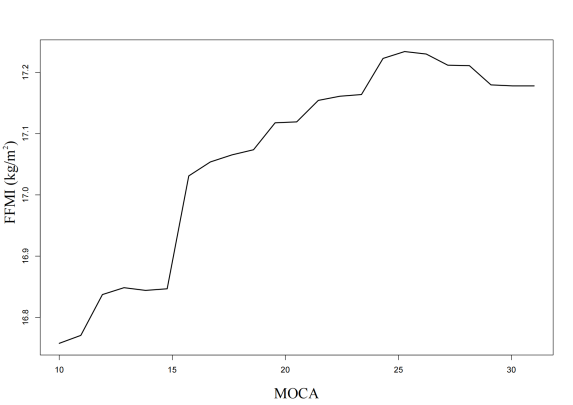 |
| **Figure S4.** Partial dependence plot (PDP) of the random forest model in the prediction of FFMI. PDP demonstrates the marginal effect of lecithin in the prediction of FFMI in the (a) second follow-up visit and (b) third follow-up visit data. PDP demonstrates the marginal effect of MoCA in the prediction of FFMI in the (c) second follow-up visit and (d) third follow-up visit data. | |

| a | b | c | d |
| --- | --- | --- | --- |
| 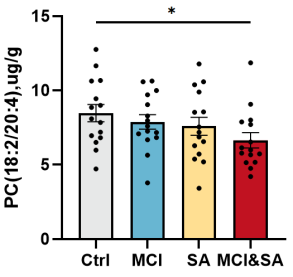 | 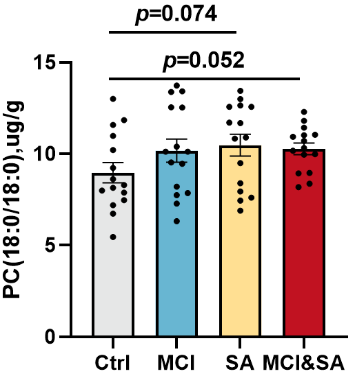 | 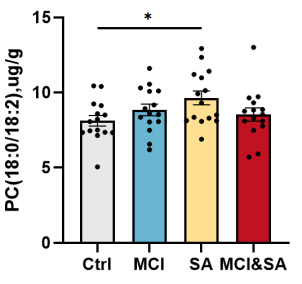 | 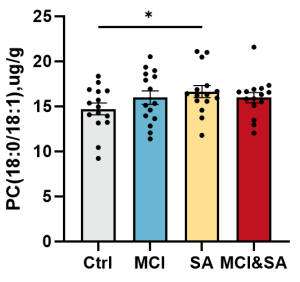 |
| e | f | g | h |
| 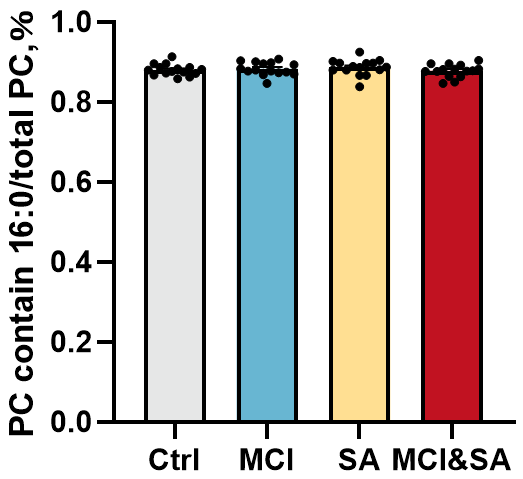 | 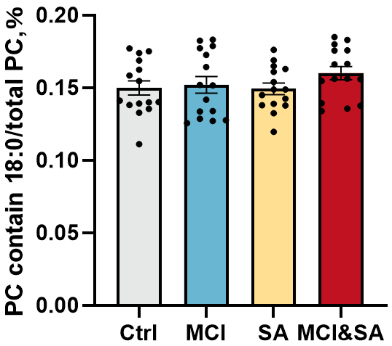 | 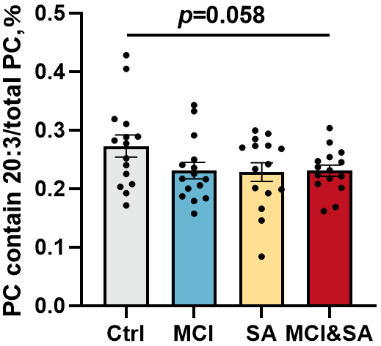 | 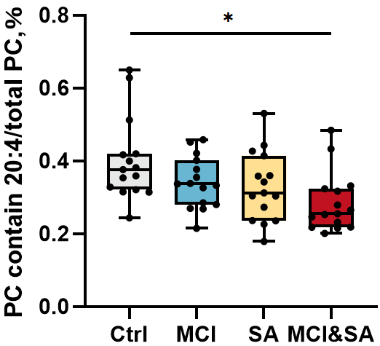 |
| **Figure S5.** Targeted lipidomics among control, MCI, SA and MCI&SA groups. a, PC (18:2/20:4) containing more polyunsaturated fatty acid in the MCI&SA group. b-d, PC (18:0/18:0) (b) , PC (18:0/18:2) (c) and PC (18:0/18:1) (d)containing more unsaturated fatty acids in the SA group. e, f. Percentage of PC containing 16:0 (e), 18:0 (f) in the total expression of PC. g, h. The percentage of PC with 20:3 (g) and 20:4 (h) was significantly lower in the MCI&SA group. n = 15 per group. Data in all line or bar graphs are shown as mean ± s.e.m. For the box-and whiskers-graphs, minima, maxima, median, 25th and 75th percentiles are shown, with whiskers indicating smallest and largest values. Two-tailed Student’s t-tests, Welch’s t-tests and Mann-Whitney U tests were performed. *P < 0.05. | | | |

| a | b |
| --- | --- |
| **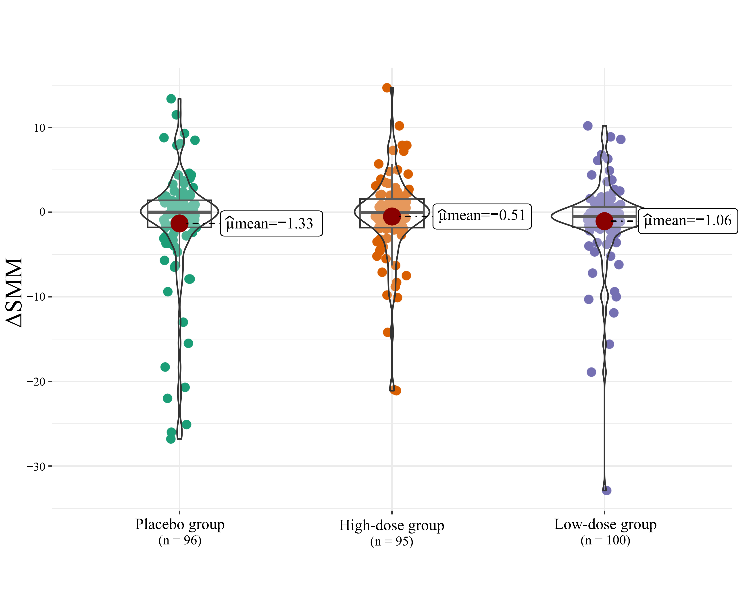** | 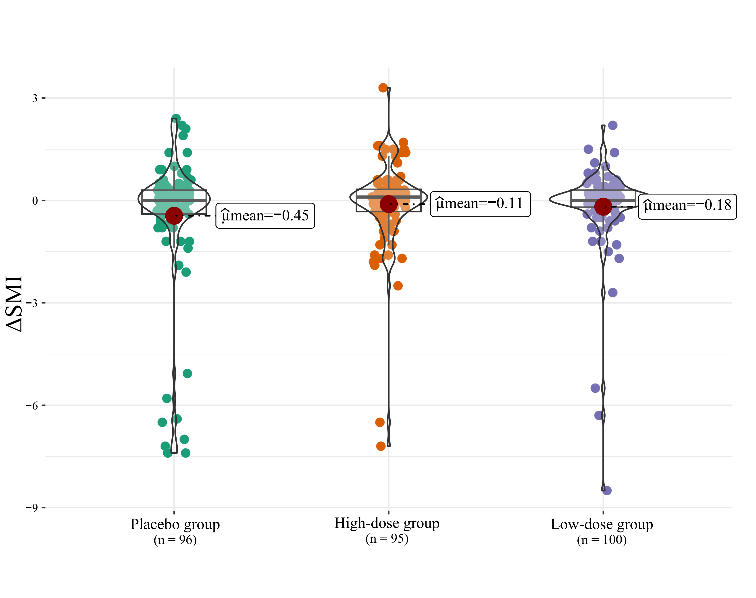 |
| c |  |
| **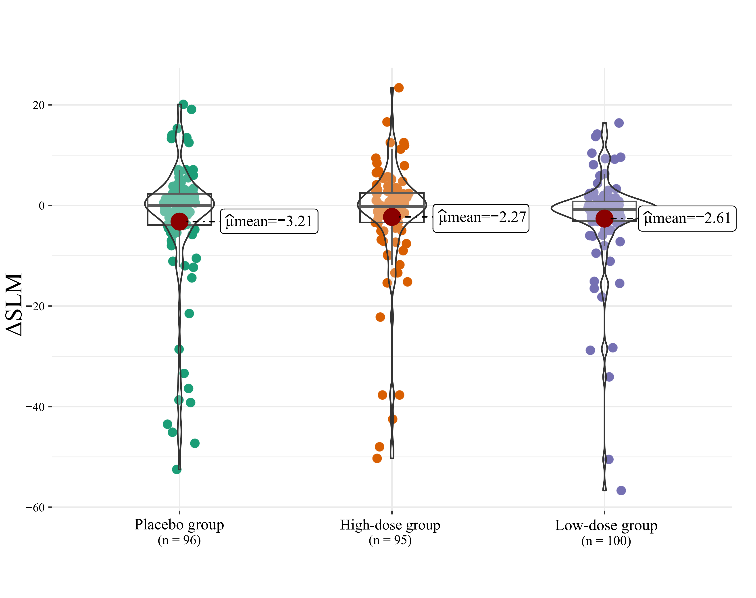** |  |
| **Figure S6.** (a) Comparisons of ΔSMM pre- and post-intervention among palcebo, high-dose and low-dose groups. (b) Comparisons of ΔSMI pre- and post-intervention among palcebo, high-dose and low-dose groups. (c) Comparisons of ΔSLM pre- and post-intervention among palcebo, high-dose and low-dose groups. | |

| a |  | | | | b | | | | | | | | |  |
| --- | --- | --- | --- | --- | --- | --- | --- | --- | --- | --- | --- | --- | --- | --- |
| 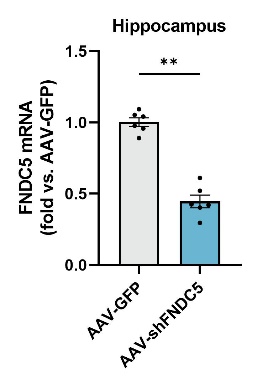 | 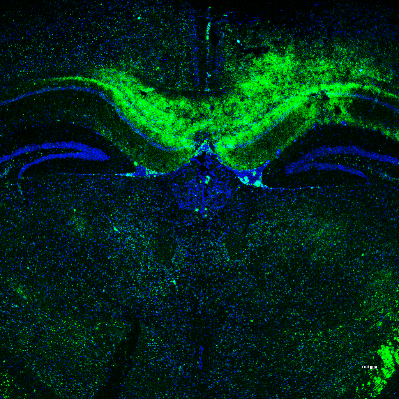 | | | | 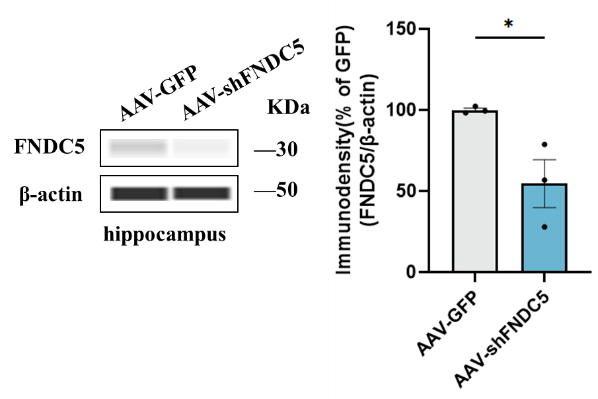 | | | | | | | | |  |
| c | | | | d | | | | e | | | | f | | |
| 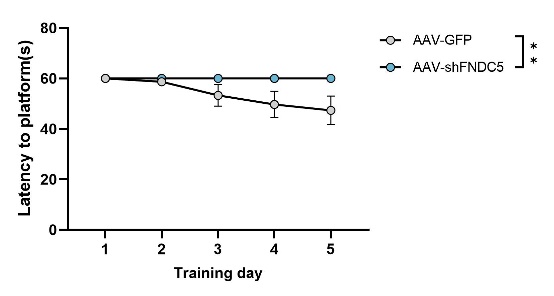 | | | | 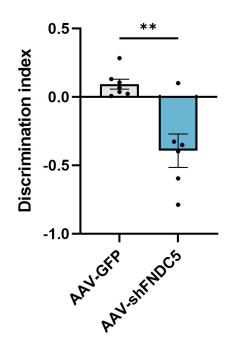 | | | | 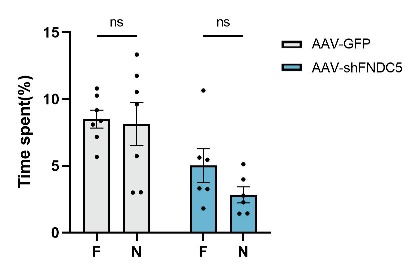 | | | | 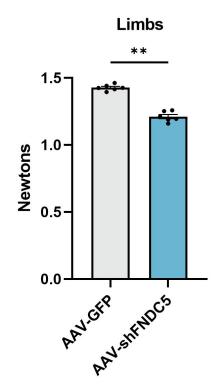 | | |
| g | h | | i | | | | | | j | k | | | |  |
| 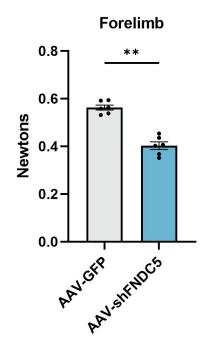 | 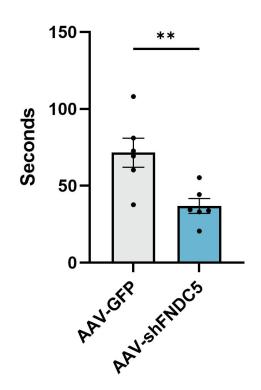 | | 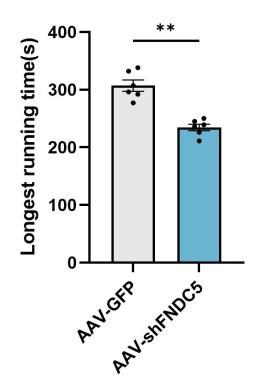 | | | | | | 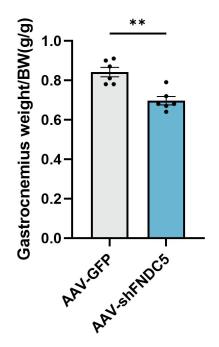 | 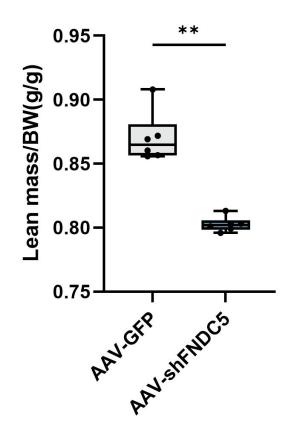 | | | |  |
| l | | m | | | | n | | | | | | o | |  |
| 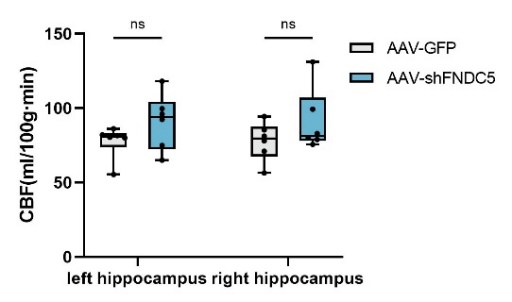 | | 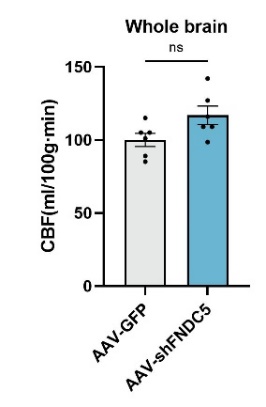 | | | | 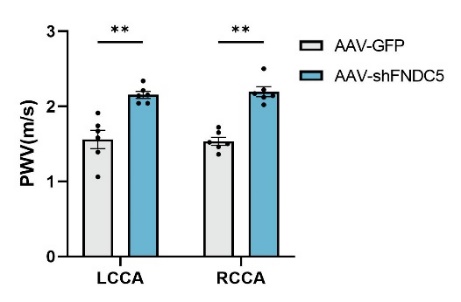 | | | | | | 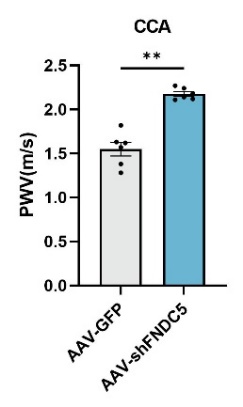 | |  |
| p | | q | | | | | r | | | |  | | |  |
| 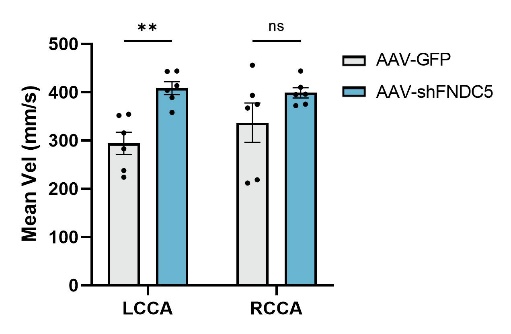 | | 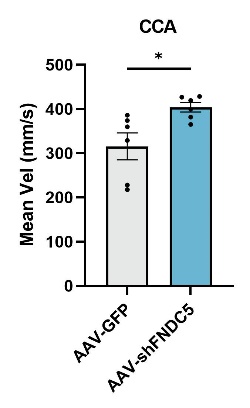 | | | | | 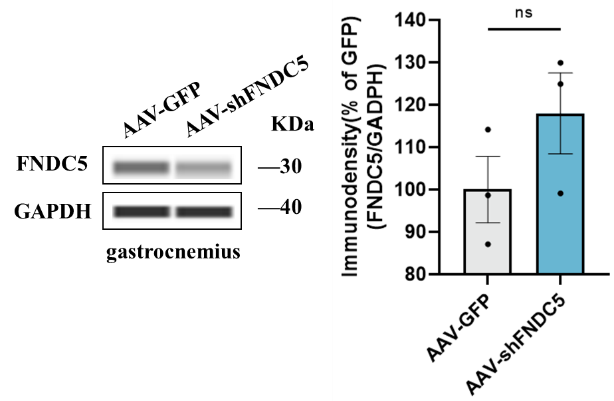 | | | | | | |  |
| s | t | | u | | | | | | |  | | | |  |
| 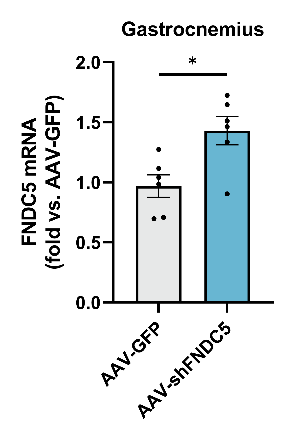 | 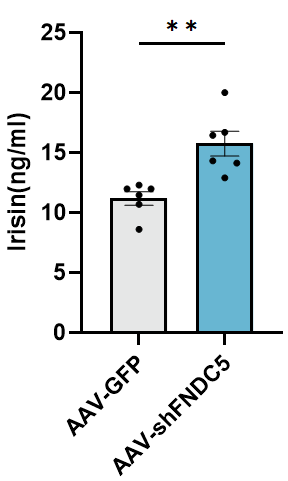 | | 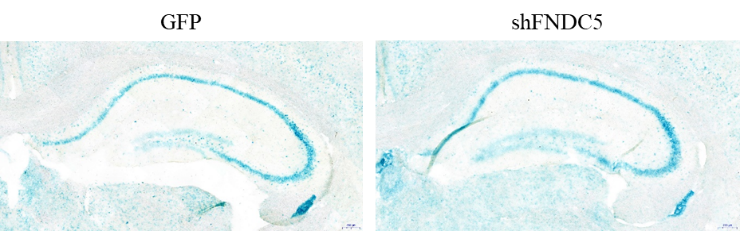 | | | | | | | | | | 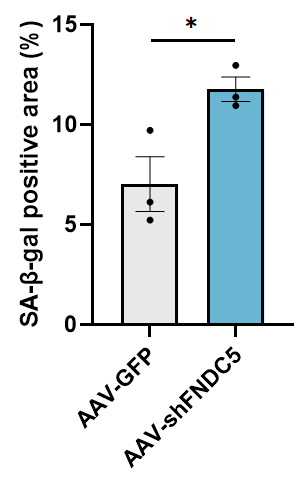 |  |
| v | | | | | | | | | | | | | |  |
| 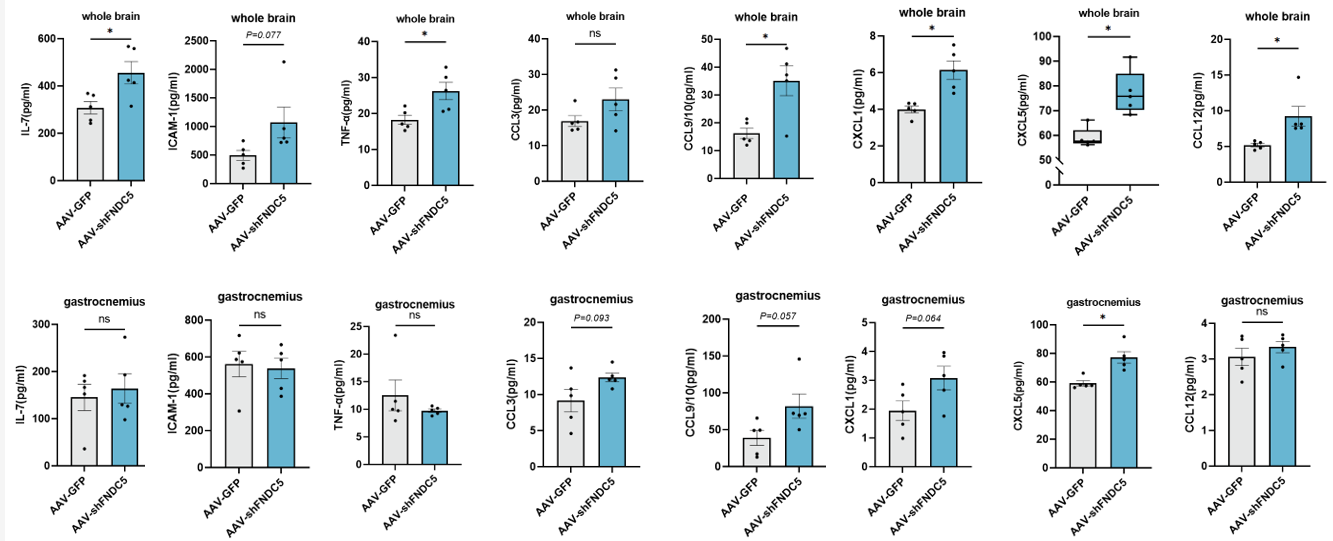 | | | | | | | | | | | | | |  |
| **Figure S7.** Downregulation of brain FNDC5/irisin impairs memory learning and muscle function in mice. a and b, Levels of FNDC5 mRNA (a) and FNDC5/irisin protein (b) in control (AAV-GFP) compared to AAV-shFNDC5-injected mice. c, latency to reach target platform of MWM. n = 6 per group. d and e, DI (d) of NOR and exploration time for familiar (F) and new objects (N) (e). AAV-GFP, n = 7, AAV-shFNDC5, n = 6. f and g, Grip strength of limbs (f) and forelimb (g). h, Hanging grid test. i, Rotarod. j, Weights of gastrocnemius muscles. k, lean mass. n = 6 per group. l and m, Hippocampus (l) and whole brain (m) of CBF. n-q, PWV (n, o) and blood flow velocity (p, q) in both carotid arteries. n = 6 per group. r and s, Protein levels (r) and mRNA levels (s) of FNDC5 in gastrocnemius. n = 6 independent tissue donors for mRNA; 3 donors for protein levels. t, Plasma irisin levels. n = 6 per group. u, Representative images and quantitative analysis of SA-β-gal staining of hippocampus (5×, scale bar = 200 μm; n = 3). v, Inflammation levels in the hippocampus and muscles. n = 5 per group. Data in all line or bar graphs are shown as mean ± s.e.m. For the box-and whiskers-graphs, minima, maxima, median, 25th and 75th percentiles are shown, with whiskers indicating smallest and largest values. Two-tailed Student’s t-tests, Welch’s t-tests and Mann-Whitney U tests were performed. *P < 0.05; **P < 0.01. | | | | | | | | | | | | | |  |

| a | |  | | | | | b | | | | | | | | |  |
| --- | --- | --- | --- | --- | --- | --- | --- | --- | --- | --- | --- | --- | --- | --- | --- | --- |
| 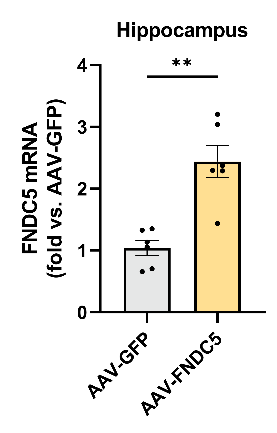 | | 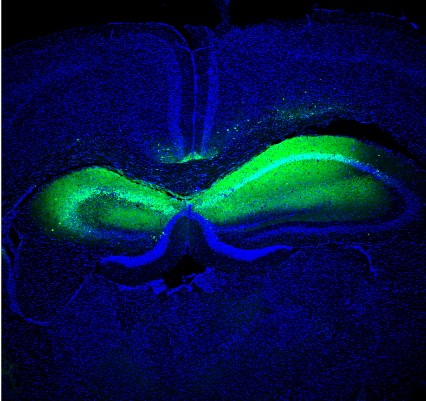 | | | | | 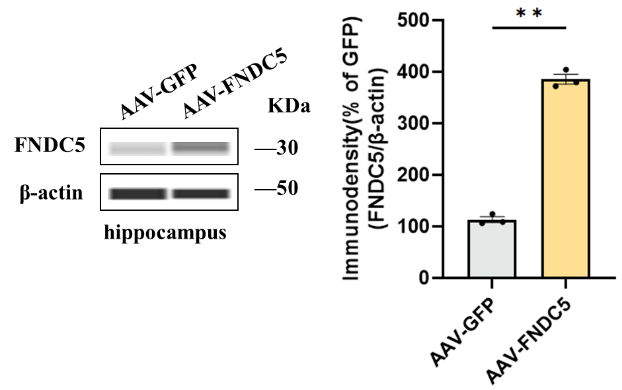 | | | | | | | | |  |
| c | | | | d | | | | e | | | | | | f | | |
| 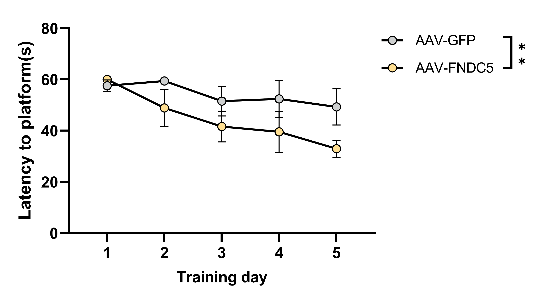 | | | | 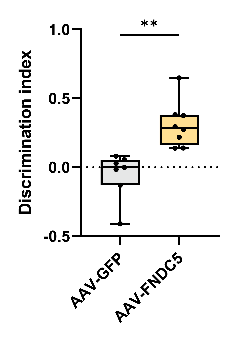 | | | | 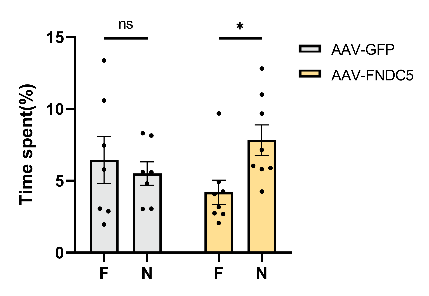 | | | | | | 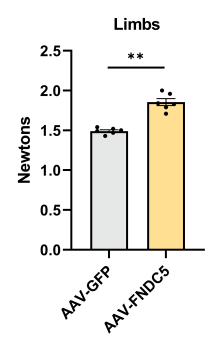 | | |
| g | | | h | | | i | | | | | j | | | | |  |
| 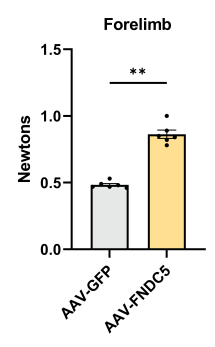 | | | 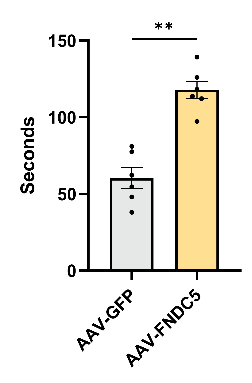 | | | 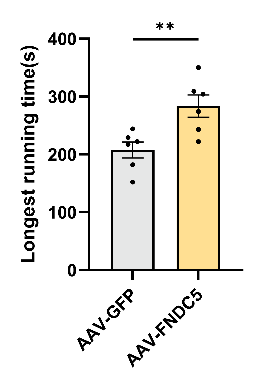 | | | | | 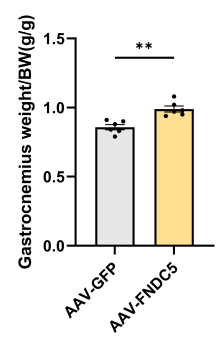 | | | | |  |
| k | | | | l | | | m | | | | | | | n | |  |
| 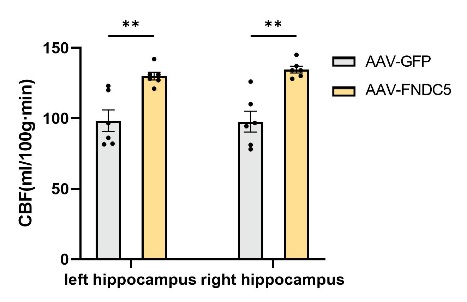 | | | | 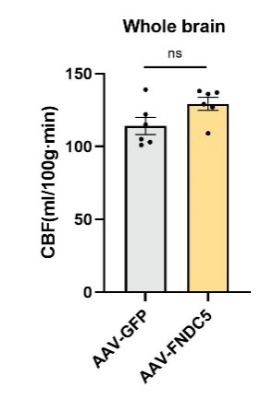 | | | 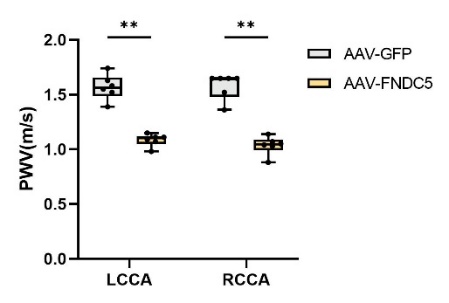 | | | | | | | 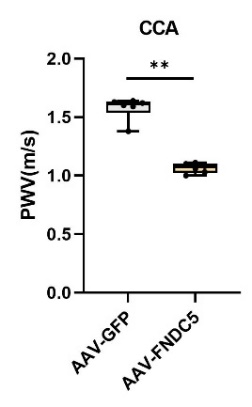 | |  |
| o | | | | p | | | | | q | | | |  | | |  |
| 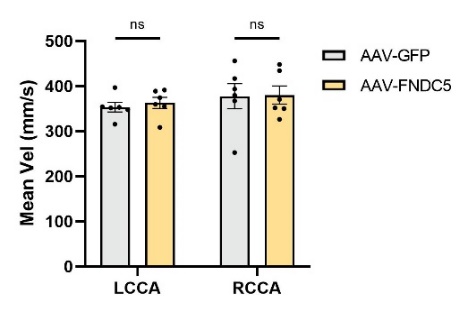 | | | | 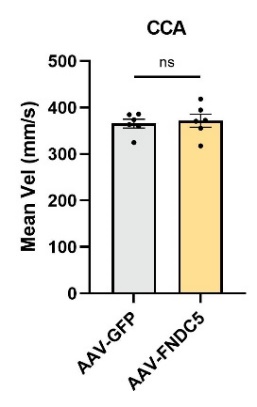 | | | | | 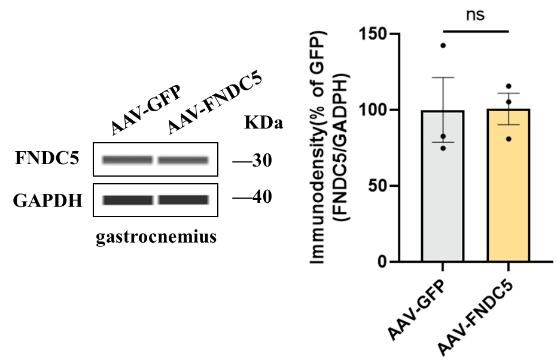 | | | | | | |  |
| r | s | | | | t | | | | |  | | | | |  |  |
| 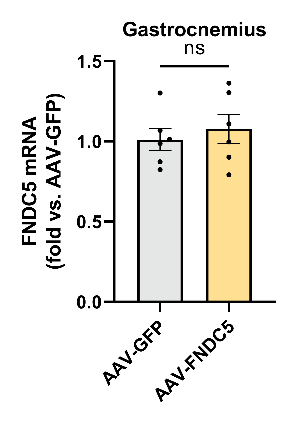 | 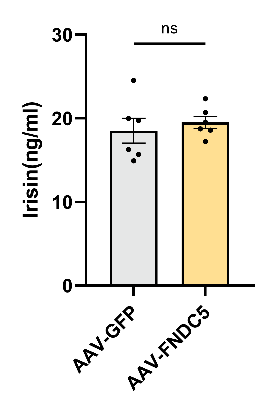 | | | | 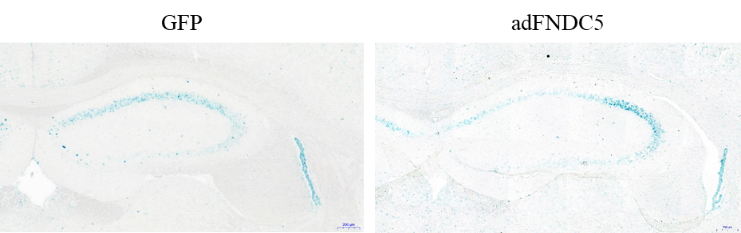 | | | | | | | | | | 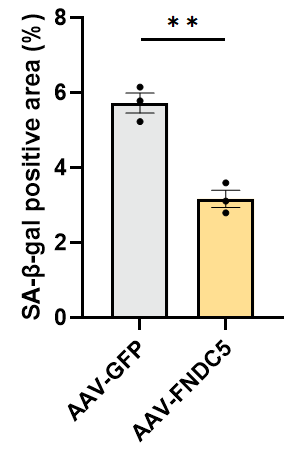 |  |
| u |  | | | |  | | | | |  | |  | | | |  |
| 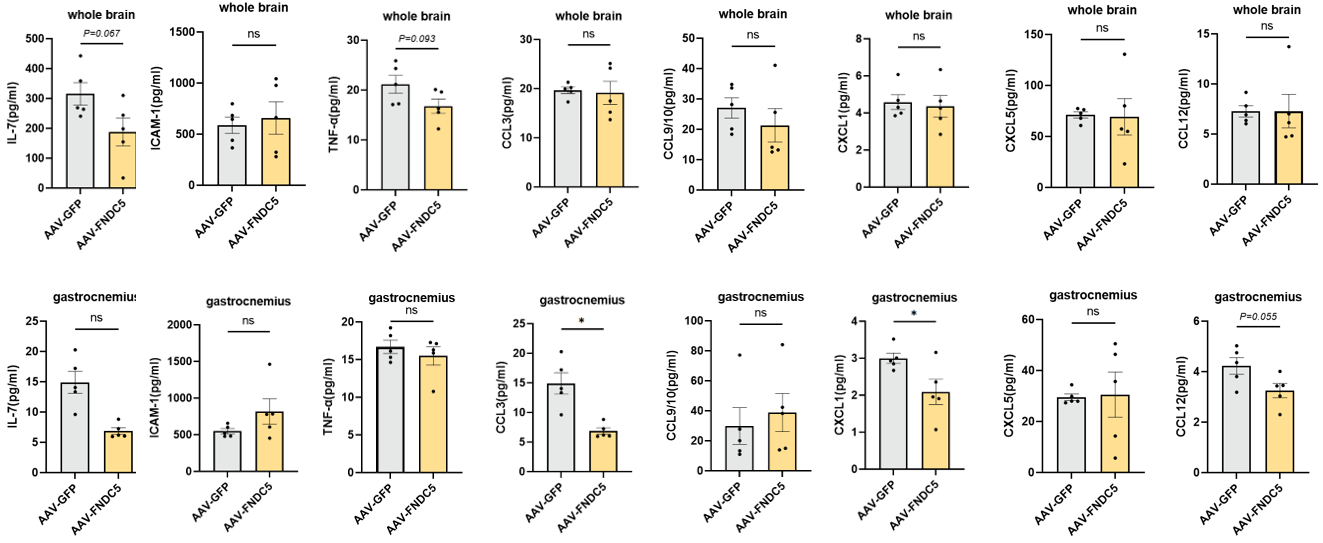 | | | | | | | | | | | | | | | |  |
| **Figure S8.** Administration of AAV-FNDC5 in the brain alleviates learning memory impairment and muscle decay. a and b, Levels of FNDC5 mRNA (a) and FNDC5/irisin protein (b) in control (AAV-GFP) compared to AAV-FNDC5-injected mice. c, latency to reach target platform of MWM. n = 6 per group. d and e, DI (d) of NOR and exploration time for familiar (F) and new objects (N) (e). AAV-GFP, n = 7, AAV-FNDC5, n = 8. f and g, Grip strength of limbs (f) and forelimb (g). h, Hanging grid test. i, Rotarod. j, Weights of gastrocnemius muscles. n = 6 per group. k and l, Hippocampus (k) and whole brain (l) of CBF. m-p, PWV (m, n) and Blood flow velocity (o, p) in both carotid arteries. n = 6 per group. q and r, Protein levels (q) and mRNA levels (r) of FNDC5 in gastrocnemius. n = 6 independent tissue donors for mRNA; 3 donors for protein levels. s, Plasma irisin levels. n = 6 per group. t, Representative images and quantitative analysis of SA-β-gal staining of hippocampus (5×, scale bar = 200 μm; n = 3). u, Inflammation levels in the hippocampus and muscles. n = 5 per group. Data in all line or bar graphs are shown as mean ± s.e.m. For the box-and whiskers-graphs, minima, maxima, median, 25th and 75th percentiles are shown, with whiskers indicating smallest and largest values. Two-tailed Student’s t-tests, Welch’s t-tests and Mann-Whitney U tests were performed. *P < 0.05; **P < 0.01. | | | | | | | | | | | | | | | |  |

| a | b | c | d | e |
| --- | --- | --- | --- | --- |
| 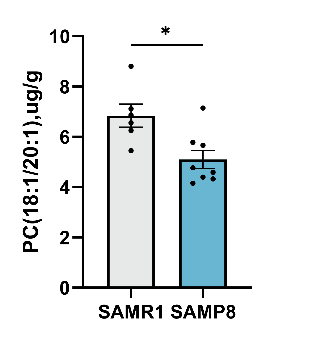 | 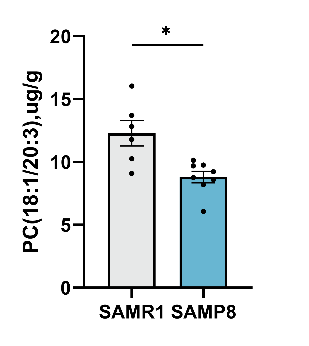 | 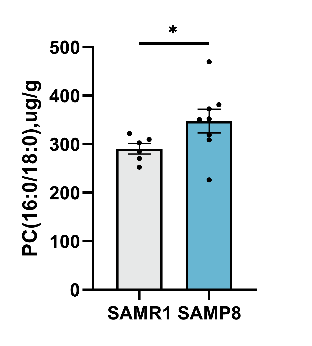 | 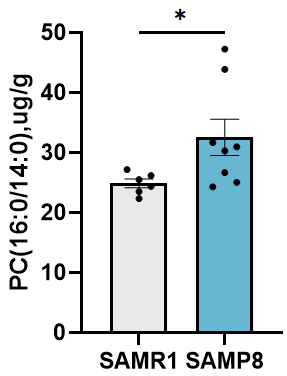 | 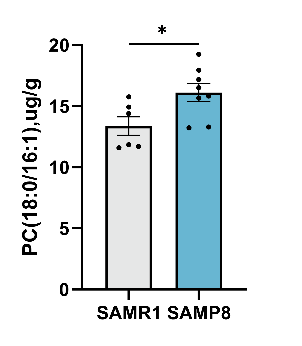 |
| f | g | h |  |  |
| 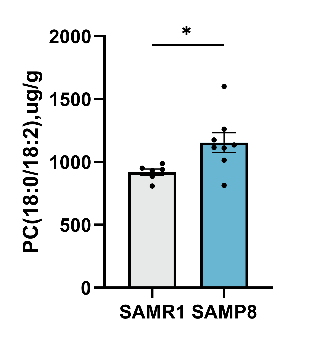 | 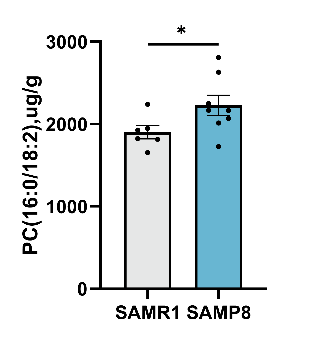 | 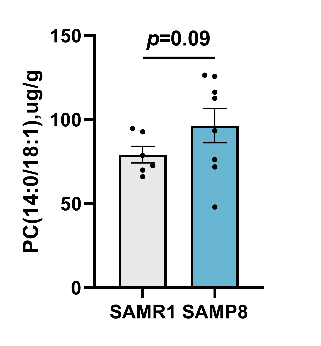 |  |  |
| **Figure S9.** Targeted lipidomic analysis in SAMP8 mice. a, b. PC (18:1/20:1) (a) and PC (18:1/20:3) (b) containing more unsaturated fatty acids in mouse erythrocytes. c-h, PC (16:0/18:0) (c), PC (16:0/14:0) (d), PC (18:0/16:1) (e), PC (18:0/18:2) (f), PC (16:0/18:2) (g) and PC (14:0/18:1) (h) which contained more saturated fatty acids in mouse erythrocytes. SAMR1, n = 6, SAMP8, n = 8. Data presented as mean ± s.e.m. Two-tailed Student’s t-tests and Welch’s t-tests were performed. *P < 0.05; **P < 0.01. | | | | |

| a | b | c |
| --- | --- | --- |
| 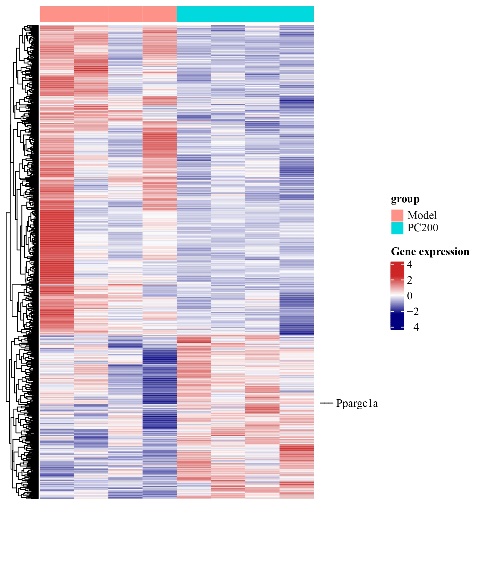 | 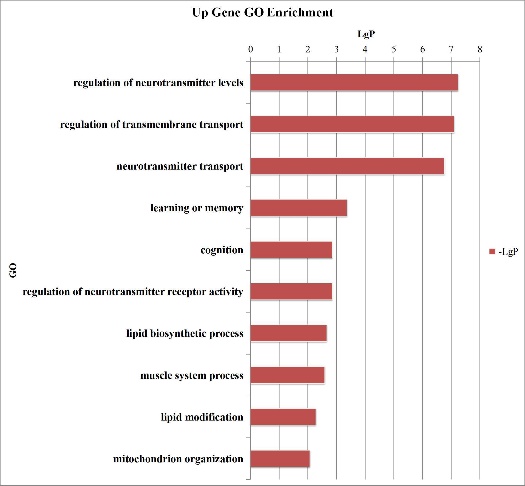 | 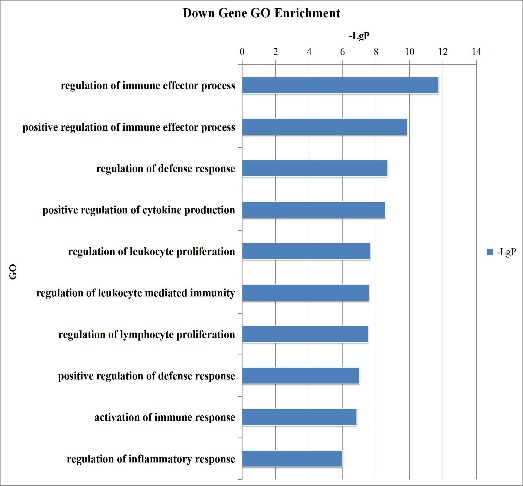 |
| d | e | f |
| 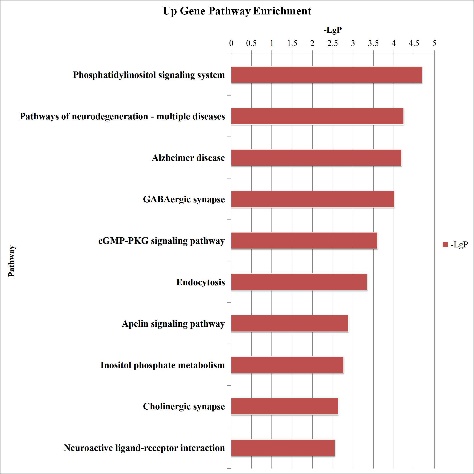 | 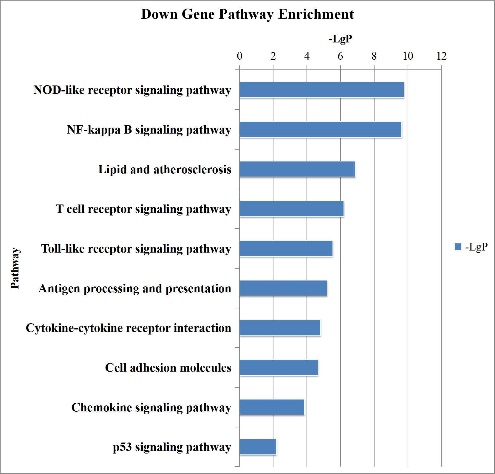 | 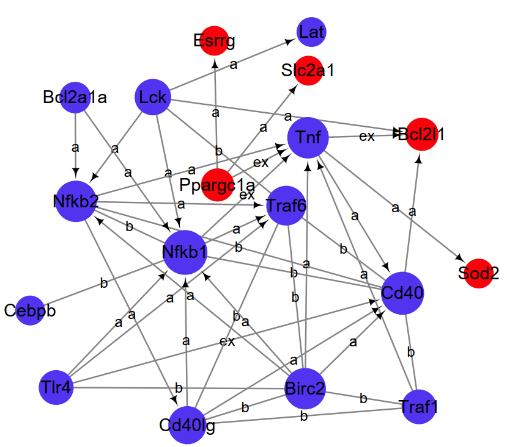 |
| **Figure S10.** Transcriptomic changes leading by PC treatment in SAMP8 mice. a, Heatmap of differentially expressed genes (DEGs) between Model group and PC200 group. Red blocks represent the overexpressed genes, while the blue blocks represent the lowly expressing genes, Ppargc1a: PGC1α. b and c, Semantic GO term enrichment analysis of upregulated (b) and downregulated (c) hippocampal genes for biological processes (BP) in SAMP8 mice by PC treatment. d-e, Upregulated (d) and downregulated (e) Kegg pathways in the hippocampus of PC200 group. f, Signal-net of PC intervention in cognitive function. The red circles represent the up-regulated genes and the blue circles represent the down-regulated genes. The area of the circle represents the degree. Interaction between the genes is shown as: a activation, b binding, ex expression. n = 4 per group.  **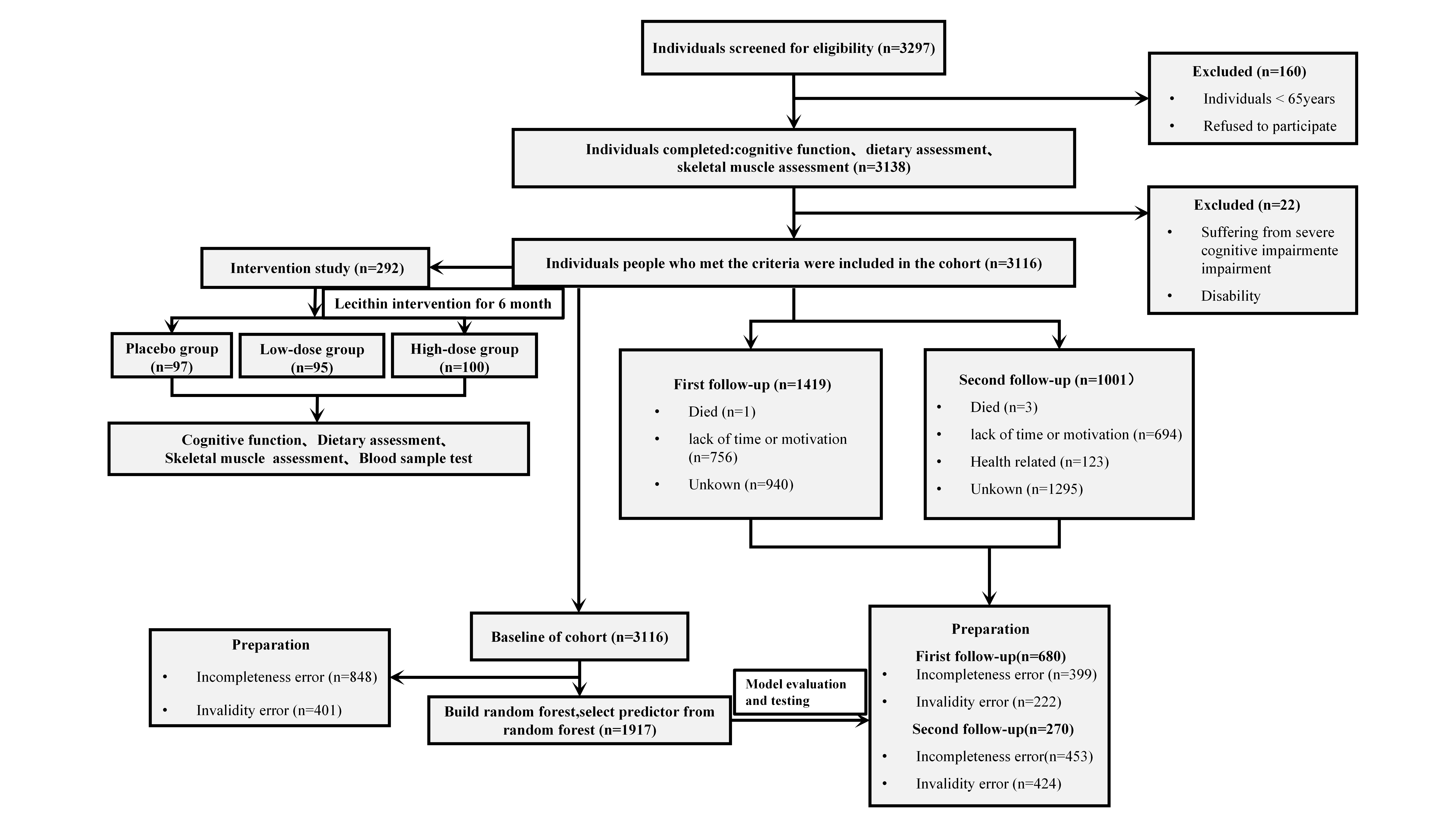** | | |

**Figure S11. Flowchart of crowd cohort**

| **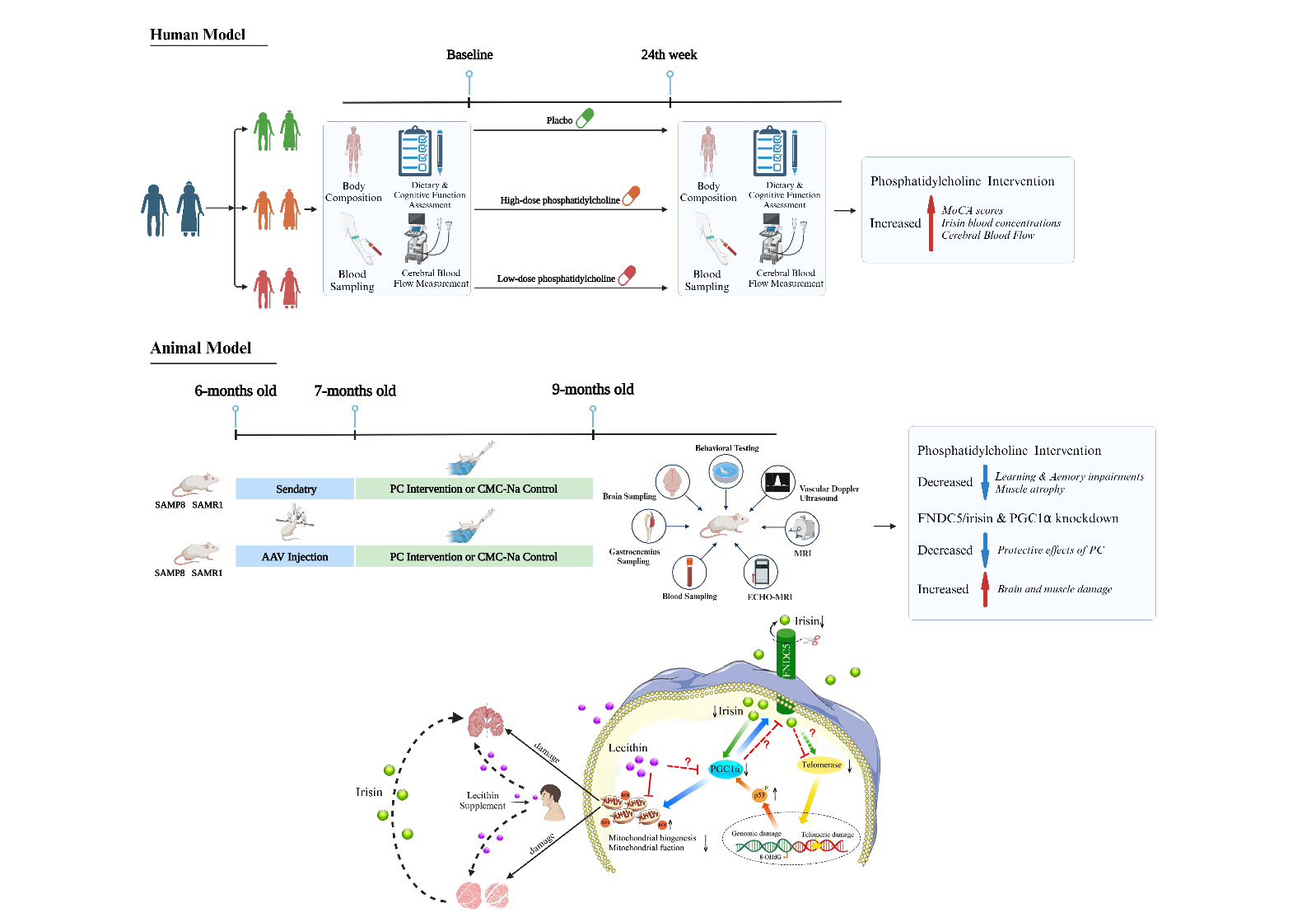** |
| --- |
| **Figure S12. An overview of the RCT study design and animal study design.** **Created with BioRender.com.** |

**Supplementary tables**

| **Table S1** Comparison of PCs contained different fatty acids in each group | | | |
| --- | --- | --- | --- |
|  | Ctrl | Model | PC 200 |
| PC containing PUFA |  |  |  |
| PC(18:1/20:3),ug/g | 12.3 ± 2.5^a)^ | 8.8 ± 1.3^a)^ | 10.1 ± 2.3 |
| PC(18:2/20:3),ug/g | 3.7 ± 0.6^a)^ | 4.5 ± 1.5 | 4.9 ± 0.8^a)^ |
| PC(18:1/20:5),ug/g | 1.4 ± 0.6^a)^ | 1.7 ± 0.7 | 2.1 ± 0.4^a)^ |
| PC(18:2/20:5),ug/g | 0.9 ± 0.2^a)^ | 1.6 ± 0.9 | 2.0 ± 0.5^a)^ |
| PC(18:2/20:2),ug/g | 2.4 ± 0.3^a)^ | 3.2 ± 0.9 | 3.4 ± 0.5^a)^ |
| PC containing SFA |  |  |  |
| PC(16:0/18:0),ug/g | 290.4 ± 25.9 | 347.7 ± 69.3*^p^*^=0.08^ | 320.3 ± 44.6 |
| PC(16:0/18:2),ug/g | 1902.0 ± 196.1^a)^ | 2229.1 ± 344.5 *^p^*^=0.06^ | 2170.7 ± 201.3^a)^ |
| PC(18:0/16:1),ug/g | 13.4 ± 1.9^a)^ | 16.1 ± 2.1^a)^ | 15.7 ± 3.4 |
| PC(18:0/18:2),ug/g | 918.1 ± 62.4^a) ,b)^ | 1153.6 ± 223.4^a)^ | 1103.6 ± 150.8^b)^ |
| PC(18:0/20:1),ug/g | 3.4 ± 0.6^a)^ | 3.0 ± 0.6 | 2.7 ± 0.3^a)^ |
| PC(20:0/18:1),ug/g | 10.7 ± 1.5^a)^ | 9.1 ± 1.9 | 7.5 ± 0.9^a)^ |
| Ctrl, n = 6, Model, n = 8, PC200, n = 6. Data are shown as mean ± s.e.m. Two-tailed Student’s t-tests, Welch’s t-tests were performed. Values with the same footnotes indicates a significant difference at p < 0.05. | | | |
